# Influence of Nanoaggregation Routes on the Structure and Thermal Behavior of Multiple-Stimuli-Responsive Micelles from Block Copolymers of Oligo(ethylene glycol) Methacrylate and the Weak Acid [2-(Hydroxyimino)aldehyde]butyl Methacrylate

**DOI:** 10.1021/acs.langmuir.2c02515

**Published:** 2022-11-08

**Authors:** Irene Antignano, Francesca D’Acunzo, Davide Arena, Stefano Casciardi, Alessandra Del Giudice, Francesca Gentile, Maria Pelosi, Giancarlo Masci, Patrizia Gentili

**Affiliations:** † Department of Chemistry, Sapienza University of Rome, P.le A. Moro 5, 00185Roma, Italy; ‡ Institute of Biological Systems (ISB), Italian National Research Council (CNR), Sezione Meccanismi di Reazione, c/o Department of Chemistry, Sapienza University of Rome, Piazzale Aldo Moro 5, 00185Roma, Italy; § National Institute for Insurance Against Accidents at Work (INAIL Research), Department of Occupational and Environmental Medicine, Epidemiology and Hygiene, Via Fontana Candida 1, 00078Monte Porzio Catone (Rome), Italy

## Abstract

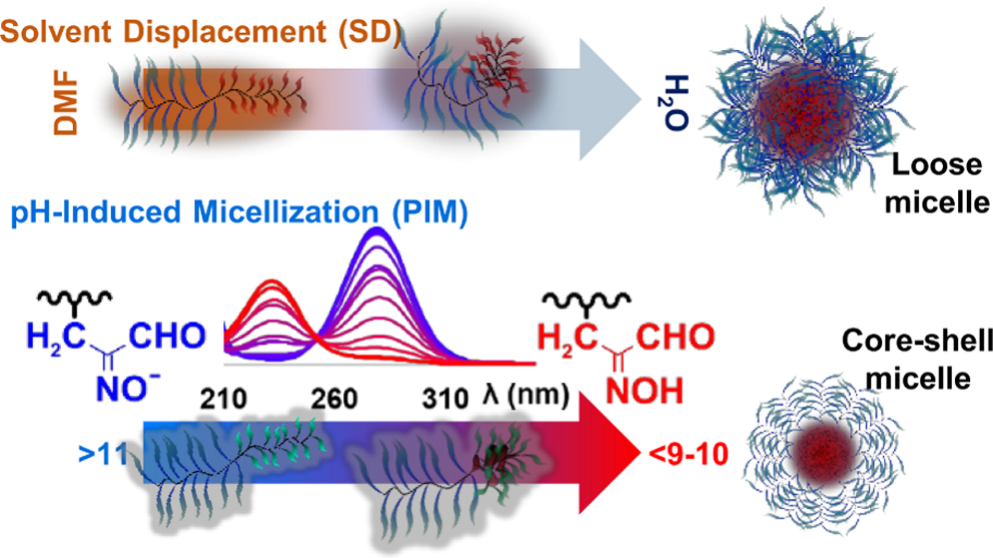

In this work, we compare nanoaggregation driven by pH-induced micellization (PIM) and by the standard solvent displacement (SD) method on a series of pH-, light-, and thermosensitive amphiphilic block copolymers. Specifically, we investigate poly(HIABMA)-*b*-poly(OEGMA) and poly(HIABMA)-*b*-poly(DEGMA-*r*-OEGMA), where HIABMA = [(hydroxyimino)aldehyde]butyl methacrylate, OEGMA = oligo(ethylene glycol)methyl ether methacrylate, and DEGMA = di(ethylene glycol)methyl ether methacrylate. The weakly acidic HIA group (p*K*_a_ ≈ 8) imparts stability to micelles at neutral pH, unlike most of the pH-responsive copolymers investigated in the literature. With SD, only some of our copolymers yield polymeric micelles (34–59 nm), and their thermoresponsivity is either poor or altogether absent. In contrast, PIM affords thermoresponsive, smaller micelles (down to 24 nm), regardless of the polymer composition. In some cases, cloud points are remarkably well defined and exhibit limited hysteresis. By combining turbidimetric, dyamic light scattering, and small-angle X-ray scattering measurements, we show that SD yields loose micelles with POEGMA segments partly involved in the formation of the hydrophobic core, whereas PIM yields more compact core–shell micelles with a well-defined PHIABMA core. We conclude that pH-based nanoaggregation provides advantages over block-selective solvation to obtain compact micelles exhibiting well-defined responses to external stimuli.

## 1 Introduction

**Scheme 1:**
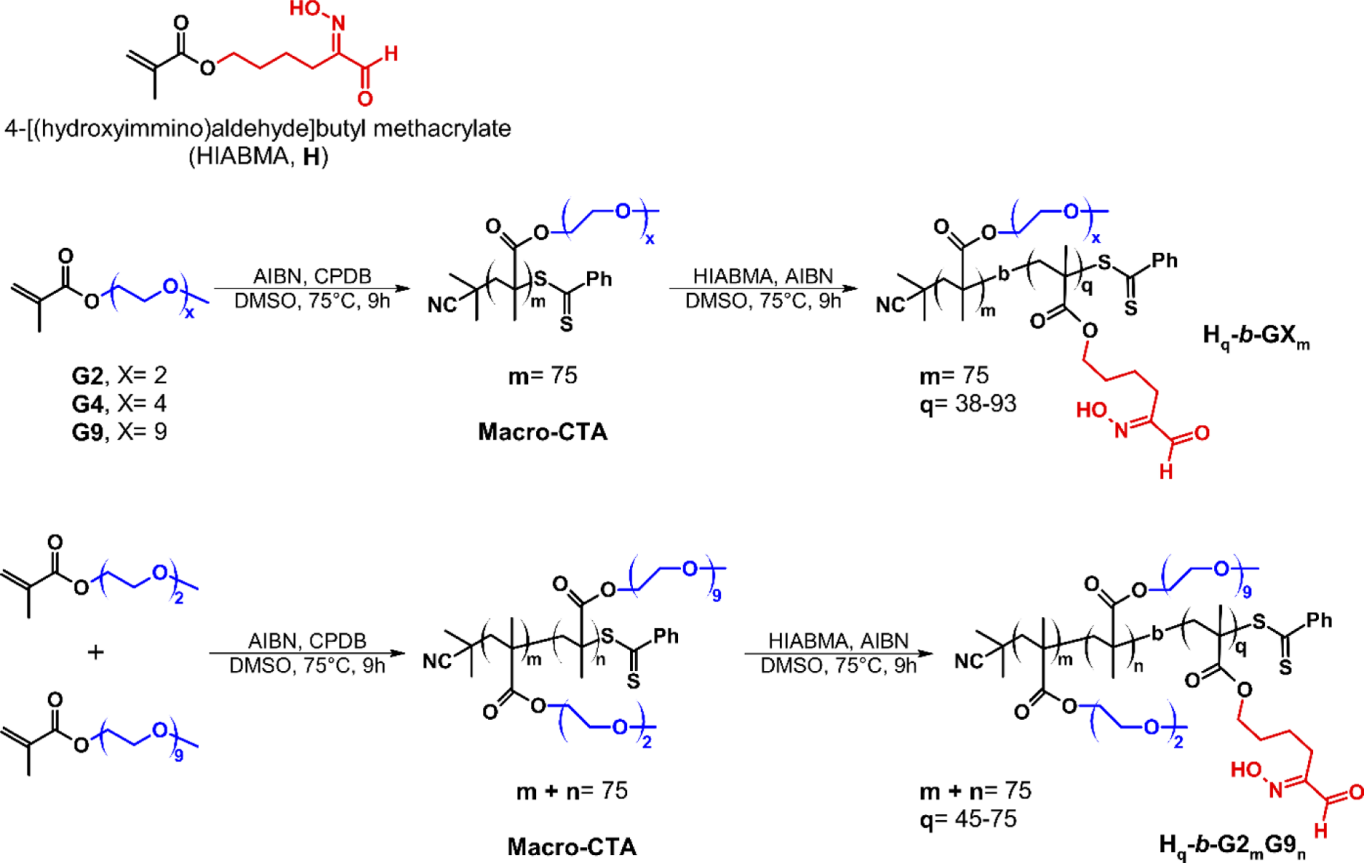
Synthesis of Poly(HIABMA)-*b*-poly[oligo(ethylene glycol)methyl ether methacrylate] (**H**_**q**_**-*b*-GX**_***m***_) or Poly(HIABMA)-*b*-poly{[di(ethylene glycol)methyl ether methacrylate]-*r*-[oligo(ethylene glycol)methyl ether methacrylate]} (**H**_**q**_**-*b*-G2**_***m***_**G9**_***n***_); [GX]/[CPDB]/[AIBN] = 75:1:0.25, [GX] = 0.8 M; [HIABMA]/[Macro-CTA]/[AIBN]= (43–100):1:0.25, [HIABMA] = 0.5–0.8 M

The development of versatile polymerization techniques triggers limitless creativity in the synthesis of macromolecules of diverse chemistry and topology, including multi-responsive copolymers.^1^ Potential applications in sensors, electronics, and biomedical fields draw attention toward the properties of polymeric nanoaggregates in connection with other interacting systems through chemical and physical signals.^2−6^ Self-assembly of surfactants and polymeric amphiphiles mostly relies on the minimization of unfavorable thermodynamic interactions involving molecular segments and solvents.^7,8^ This driving force provides the basis for solvent displacement (SD), in which assembly is induced by gradually switching from a good solvent to a segment-selective one. However, as more stimuli-responsive copolymers are being synthesized, micellization has been induced by taking advantage of alternative driving forces other than inherent solvophobicity.^9^ This opportunity has had a significant impact on the field of double-hydrophilic block copolymers.^10^ For example, Vagias et al.^11^ have recently investigated the nanoscale inner morphology of temperature-induced aggregates of PNIPAAM-*b*-POEGMA copolymers. The PNIPAAM is desolvated above its lower critical solution temperture, thereby forming the core of micellar aggregates. Remarkably, for some copolymer compositions, it appears that micelles are not disrupted upon cooling. Zeng and co-workers^12^ obtained non-covalently crosslinked double-hydrophilic polymeric micelles through multiple and complementary hydrogen bonding. Ionizable groups provide the opportunity to utilize the pH trigger to switch a copolymer block from charged to neutral, hence from hydrophilic to hydrophobic, thus enabling pH-induced aggregation or complex behaviors such as micellar inversion (“schizophrenic” micelles).^13,14^ Polyelectrolytes are weaker bases or acids than the corresponding monomers due to electrostatic repulsion of the charged groups, and ionization of each group is correlated in a complex way to the number and distribution of the other ionizable groups through conformational changes and hydrogen bonding.^15^ Furthermore, when ionizable groups are linked to a hydrophobic backbone, conformational changes and self-assembly may result from the interplay of hydrophobic and electrostatic interactions.^16−18^ It is worth noting that when pH adjustment is used to induce self-assembly, the micelles may be kinetically frozen, thanks to hydrogen bonding interactions between partially ionized groups.^19,20^ In other words, the system self-assembles into a non-equilibrium state that is dependent on the conditions leading to its formation, and the copolymers have very slow chain exchange dynamics and assemble into locally isolated, non-ergodic structures.^21,22^ Typically, pH-responsive units comprise weakly basic amino groups (e.g., 2-dialkylaminoethyl methacrylates, 2-vinylpyridine^23^) that are protonated at acidic pH,^14,16,24−28^ and weak acids (e.g. acrylic or methacrylic, vinylbenzoic) that are dissociated at basic pH.^13,18,29−31^ In these cases, cloud-point modulation and/or micelle formation, disruption, or rearrangement occur at neutral to moderately acidic or basic pH values. This is a useful feature for applications that require responsivity around physiologically relevant pH values.^32,33^ In an interesting line of investigation, pH-responsive units were revealed by photocleavage of *o*-nitrobenzyl ester units in PEG-based amphiphilic methacrylates that were therefore capable of multiple thermal micellization.^34,35^ The pH-sensitivity of a polymer could, in principle, be exploited to obtain polymeric micelles that are stable around physiological pH as an alternative to the standard solvent switch technique. To this end, ionization should occur well above or below pH 7, contrary to most of the functional groups investigated so far.^2^ pH-driven self-assembly may produce different micellar structures than block selectivity-driven aggregation,^36,37^ thereby causing differences in the response to other stimuli (e.g., temperature or light). In recent years, we have obtained a 2-(hydroxyimino)aldehyde butyl methacrylate (HIABMA) monomer, which has a p*K*_a_ of the oxime group that is at least 3 orders of magnitude higher than that of acrylic and benzoic acids (see herein), provides multiple sites for hydrogen bonding, has potential for metal chelation, and exhibits a wavelength-dependent photochemical behavior (i.e., *E*/*Z* oxime isomerism and an unusually chemoselective Norrish–Yang cyclization).^38,39^ In other words, HIABMA-based amphiphilic block copolymers are good candidates to obtain temperature- and light-sensitive copolymers that aggregate by either pH or solvent switch, potentially leading to different nanostructures with different properties that are also stable at physiological pH. The novelty of the HIABMA group with respect to monomers bearing simple aldehydes^40−46^ or oximes^47,48^ derives from the two functional groups being adjacent, which has consequences on aldehyde photochemistry, oxime acidity, and, possibly, on metal chelation (the latter is an ongoing investigation in our group).^38^ Potential applications of HIABMA copolymers are, therefore, quite different from the widely investigated polymeric prodrugs based on C=N conjugation chemistry. In our previous work, we have obtained random copolymers of HIABMA and OEGMA, and we have investigated the response of their aqueous solutions to photostimulation and to temperature changes, the latter being due to the OEGMA units.^49,50^ In the present work, we synthesize amphiphilic block copolymers of HIAMBA and OEGMA (Scheme 1) with different compositions and thereby obtain spherical polymeric micelles by two different methods, that is SD and pH variation (pH-induced micellization: PIM).

The nanoaggregates are studied via dynamic light scattering (DLS), transmission electron microscopy (TEM), and small-angle X-ray scattering (SAXS) and their response to temperature changes is investigated by DLS and turbidimetry. With this work, we show that, when applied to the polymers under investigation, the PIM method offers several advantages over SD in terms of micelle size distribution and thermal properties. Furthermore, nanoaggregates from some polymer compositions that failed to yield micelles by SD were successfully obtained by PIM.

## 2 Experimental Section

### 2.1 Materials

Methacrylic anhydride, 1,6-hexanediol, triethylamine (TEA), 4-(dimethylamino)pyridine (DMAP), *p*-toluenesulfonic acid monohydrate, pyrrolidine 99%, oligo(ethylene glycol)methyl ether methacrylate (OEGMA, number-average molecular weight *M*_*n*_ = 500 and 300 g/mol), di(ethylene glycol)methyl ether methacrylate (*M*_*n*_ = 188.22 g/mol, 95%), and 2,2′-azobis(2-methylpropionitrile) (AIBN) were purchased from Sigma-Aldrich. Ferric chloride hexahydrate (FeCl_3_·6H_2_O) was purchased from Acros Organics. The sulfur trioxide–pyridine complex (SO_3_-Pyr) was purchased from TCI Chemicals Europe. Sodium nitrite, ACS reagent grade, was purchased from Carlo Erba Reagents S.r.l., Italy. 2-Cyanopropan-2-yl dithiobenzoate (CPDB) chain-transfer agent was purchased from STREM Chemicals, Inc., BISCHHEIM, France. All solvents were purchased from Sigma-Aldrich except dimethylformamide and THF, which were procured from VWR International, Milan, Italy. Water used for cloud-point determinations was of Milli-Q grade. Chloroform-*d* was obtained from Acros Organics and dimethylsulfoxide-*d*_6_ (DMSO-*d*_6_; glass ampules) from VWR International. OEGMA and MEO_2_MA were passed through a basic alumina column to remove the inhibitor and stored at −20 °C until use. DMSO was dried over 4 Å molecular sieves and stored under argon. 0.1 M HCl was standardized using anhydrous CaCO_3_ and then used to titrate 0.1 M sodium hydroxide immediately before use.

### 2.2 Synthesis of HIABMA

HIABMA was prepared as follows as previously reported in D’Acunzo et al. *Macromol. Chem. Phys.***2019,** 220, 1900200.
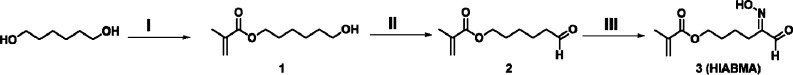


#### 2.2.1 6-Hydroxyhexyl 2-Methylprop-2-enoate (HHMA, 1)

Under a N_2_ atmosphere, Et_3_N (4.2 mL, 30 mmol), DMAP (0.73 g, 6.0 mmol), and methacrylic anhydride (4.4 mL, 30 mmol) were added to an ice-cold solution of 1,6-hexanediol (7.1 g, 60 mmol) in anhydrous THF (40 mL). The mixture was stirred at 0 °C for 1 h. MeOH (7 mL) was added, and stirring was continued for 15 min. The solvent was removed under reduced pressure, and the crude mixture was then diluted with ethyl acetate (40 mL) and washed with 1 M HCl, saturated NaHCO_3_, and brine. The organic phase was dried over Na_2_SO_4_, filtered, and solvents were removed under reduced pressure. 3.9 g of a mixture of ester **1** and dimethacrylate byproduct was obtained and used in the next step without further purification.

#### 2.2.2 6-Oxohexyl 2-Methylprop-2-enoate (OHMA, 2)

Under a N_2_ atmosphere, TEA (11 mL, 79 mmol) was added to the crude mixture, obtained in step I (3.9 g, 16 mmol of **1**), in dichloromethane (100 mL). The resulting mixture was cooled to 0 °C, a solution of SO_3_–Pyr complex (9.9 g, 62 mmol) in DMSO (65 mL) was then added and stirred; stirring was maintained for 20 min at 0 °C then for 2 h at room temperature. The crude mixture was washed with saturated NH_4_Cl, saturated NaHCO_3_, and water. The solvent was removed under reduced pressure, the mixture was re-dissolved with Et_2_O (40 mL), and then washed with brine and water. The organic phase was dried over Na_2_SO_4_, filtered, and the solvents evaporated under reduced pressure. The residue was purified by silica gel chromatography with a hexane/ethyl acetate gradient (from 30:1 to 10:1 vol/vol) to afford aldehyde **2** (1.7 g, 9.4 mmol, 59% yield).

#### 2.2.3 4-[(Hydroxyimino)aldehyde]butyl Methacrylate (HIABMA, 3)

To a solution of *p*-TSA (0.36 g, 1.9 mmol) in dimethylformamide (DMF) (12 mL), pyrrolidine (0.16 mL, 1.9 mmol) and aldehyde **2** (1.7 g, 9.4 mmol) were added. After cooling the mixture to 0 °C for 5 min, NaNO_2_ (0.65 g, 9.4 mmol) and FeCl_3_·6H_2_O (2.5 g, 9.4 mmol) were added in small aliquots. The mixture was stirred at room temperature for 5 h. Ethyl acetate (40 mL) and saturated NH_4_Cl (20 mL) were added and stirring was maintained for 30 min. The organic layer was separated, and the aqueous phase was extracted twice more with ethyl acetate. The combined organic extracts were washed with brine, dried over Na_2_SO_4_, filtered, and solvents removed under reduced pressure. Most of the reaction byproducts (mainly iron salts) were removed through first column chromatography with 50:1 silica gel excess (w/w), using a hexane/ethyl acetate gradient (20:1 to 5:1 vol/vol). Second chromatographic purification on a 100× weight excess silica gel with a dichloromethane/hexane/ethyl acetate eluent was necessary to achieve high purity [>99% by high-performance liquid chromatography (HPLC)].^24^ Dichloromethane was kept at 50% by volume throughout the elution, while the proportion of hexane and ethyl acetate was varied from 20:1 to 5:1 to afford HIABMA **3** (0.99 g, 4.6 mmol, 49% yield).

### 2.3 Synthesis of Homopolymers and Random Copolymers (Scheme 1)

The polymers were prepared by reversible addition–fragmentation chain-transfer polymerization (RAFT), using CPDB as a chain-transfer agent and initiated by AIBN. The polymerization was carried out using [monomer/s]/[CPDB]/[AIBN] = 75:1:0.25 and [monomer/s] = 0.8 M. The monomers (**G2**, **G4**, **G9**, or a mixture of **G2** and **G9**, 2 mmol in total), CPDB (5.9 mg, 27 μmol), and AIBN (1.1 mg, 6.7 μmol) were charged in a glass ampule, and the volume was adjusted with DMSO to 2.5 mL. The solution was then purged with argon for 50 min. Then, the glass ampule was flame-sealed against argon current, and the mixture was maintained at 75 °C for 9 h. The reaction was quenched by exposure to air. Proton nuclear magnetic resonance (^1^H NMR) spectroscopy and gel permeation chromatography (GPC) analyses were performed on aliquots of the polymerization crude mixture after dilution in appropriate solvents (CDCl_3_ and 0.1% DMF/LiBr, respectively) to calculate NMR-based conversions (>95%, Table 1) and *M*_*n*_ and molecular-weight dispersity (*Đ*) by GPC (Table 1). The known amount of polymer thus obtained was drawn and used without purification as macro-CTA for obtaining block copolymers by chain extension. The fraction of the crude mixture not used for chain extension was dialyzed (Spectra/Por 7 MWCO: 1000 Da cut-off dialysis tubing) against distilled water (48 h) to remove DMSO, and the pink oily product was obtained by lyophilization for further experiments.

**Table 1:** Characterization of the Polymers Obtained in This Study by RAFT Polymerization

polymer	conv. % (NMR)^a^	*M*_*n*_ (GPC) g mol^–1^ × 10^–3^	*M*_*n*_ (calc)^b^g mol^–1^ × 10^–3^	*Đ*(GPC) *M*_w_/*M*_*n*_
**G2**_**75**_	>95	8.2	14.1	1.08
**H**_**66**_**-*b*-G2**_**75**_	88	15.7	28.2	1.17
**G4**_**75**_	>95	11.0	22.5	1.12
**H**_**38**_**-*b*-G4**_**75**_	92	16.3	30.6	1.19
**H**_**73**_**-*b*-G4**_**75**_	73	18.6	38.0	1.20
**G9**_**75**_	>95	13.7	37.5	1.13
**H**_**93**_**-*b*-G9**_**75**_	93	23.3	57.3	1.15
**G2**_**69**_**G9**_**6**_	>95	8.8	16.0	1.09
**H**_**75**_**-*b*-G2**_**69**_**G9**_**6**_	>95	15.8	32.0	1.13
**G2**_**64**_**G9**_**11**_	>95	9.3	17.5	1.09
**H**_**68**_**-*b*-G2**_**64**_**G9**_**11**_	90	16.0	32.0	1.14
**G2**_**53**_**G9**_**22**_	>95	10.0	21.0	1.08
**H**_**45**_**-*b*-G2**_**53**_**G9**_**22**_	89	15.9	30.6	1.15
**H**_**60**_**-*b*-G2**_**53**_**G9**_**22**_	80	15.5	33.8	1.15
**G2**_**22**_**G9**_**53**_	>95	12.2	30.6	1.11
**H**_**67**_**-*b*-G2**_**22**_**G9**_**53**_	89	17.7	44.9	1.17

a Conversion for OEGMA polymerization or chain extension where applicable. NMR conversion values are calculated as follows: , where *I*_0_ and *I*_*t*_ are the intensities of the vinyl signals from monomers at time = 0 and *t*, relative to the terminal OEGMA signal −OCH_3_ (3H).

b , where[**M**]_0,**TOT**_ = sum of all monomers; .

### 2.4 Synthesis of Block Copolymers

The homopolymers and random copolymers described above were used as macro-CTAs in the synthesis of block copolymers by extending the chain with the HIABMA monomer using AIBN as an initiator (Scheme 1). Each crude mixture of macro-CTA was used directly for the chain extension without further purification. The macro-CTA concentration was 0.01067 M where not specified. The polymerizations were carried out using [HIABMA]/[macro-CTA]/[AIBN] = 43–100:1:0.25. The reagents were charged in a glass ampule that was flame-sealed against argon current, and the mixture was maintained at 75 °C for 9 h. The reaction was quenched by exposure to air.

#### 2.4.1 **H**_**73**_**-*b*-G4**_**75**_

HIABMA (75 mg, 0.35 mmol), **G4**_**75**_ or macro-CTA (0.33 mL, 3.5 μmol), AIBN (0.14 mg, 8.8 μmol), and DMSO (0.37 mL).

#### 2.4.2 **H**_**93**_**-*b*-G9**_**75**_

HIABMA (0.17 g, 0.80 mmol), **G9**_**75**_ or macro-CTA (0.75 mL, 8.0 μmol), AIBN (0.33 mg, 2.0 μmol), and DMSO (0.25 mL).

#### 2.4.3 **H**_**45**_**-*b*-G2**_**53**_**G9**_**22**_

HIABMA (0.13 g, 0.60 mmol), **G2**_**53**_**G9**_**22**_ or macro-CTA (1.1 mL, 12 μmol), and AIBN (0.49 mg, 3.0 μmol).

#### 2.4.4 **H**_**38**_**-*b*-G4**_**75**_

HIABMA (0.13 g, 0.60 mmol), **G4**_**75**_ or macro-CTA (*C* = 0.013 M, 1.1 mL, 14 μmol), and AIBN (0.62 mg, 3.8 μmol).

#### 2.4.5 **H**_**66**_**-*b*-G2**_**75**_

HIABMA (85 mg, 0.40 mmol), **G2**_**75**_ or macro-CTA (0.50 mL, 5.3 μmol), and AIBN (0.22 mg, 1.3 μmol).

#### 2.4.6 **H**_**75**_**-*b*-G2**_**69**_**G9**_**6**_, **H**_**68**_**-*b*-G2**_**64**_**G9**_**11**_, **H**_**60**_**-*b*-G2**_**53**_**G9**_**22**_, and **H**_**67**_**-*b*-G2**_**22**_**G9**_**53**_

HIABMA (0.17 g, 0.80 mmol), macro-CTA (1.0 mL, 11 μmol), and AIBN (0.44 mg, 2.7 μmol).

The monomer conversion (73% to >95% range, Table 1) was calculated by ^1^H NMR analysis of the crude mixture in CDCl_3_.

The pink oily polymers were obtained by dialysis (48 h) against ethyl acetate. The products were then dried under high vacuum at room temperature for 7 days. The purified products were characterized by ^1^H NMR and GPC (*M*_*n*_ and Đ, Table 1 and Figures S1–S3).

### 2.5 Preparation of Nanoaggregates

#### 2.5.1 SD Method

10 mg of polymer was solubilized in 1 mL of DMF in a quartz cuvette (1 cm optical path) and deionized water was added at 6.5 μL/min rate using a syringe pump (Thermo Electron Orion M365 Sage) with constant magnetic stirring. The transmittance at 700 nm and 25 °C [Cary 300 Bio UV–visible spectrophotometer (Varian)] was measured along the water addition process until a final solution having a H_2_O/DMF (w/w) ratio of 4:1 was obtained. Then, the solution was dialyzed (Spectra/Por 7 MWCO: 1000 Da cut-off dialysis tubing) against distilled water (48 h) to remove DMF. The volume of the final dialysate was adjusted with Milli-Q water in order to obtain the same concentration for all polymers (1.5 mg/mL).

**Figure 1:**
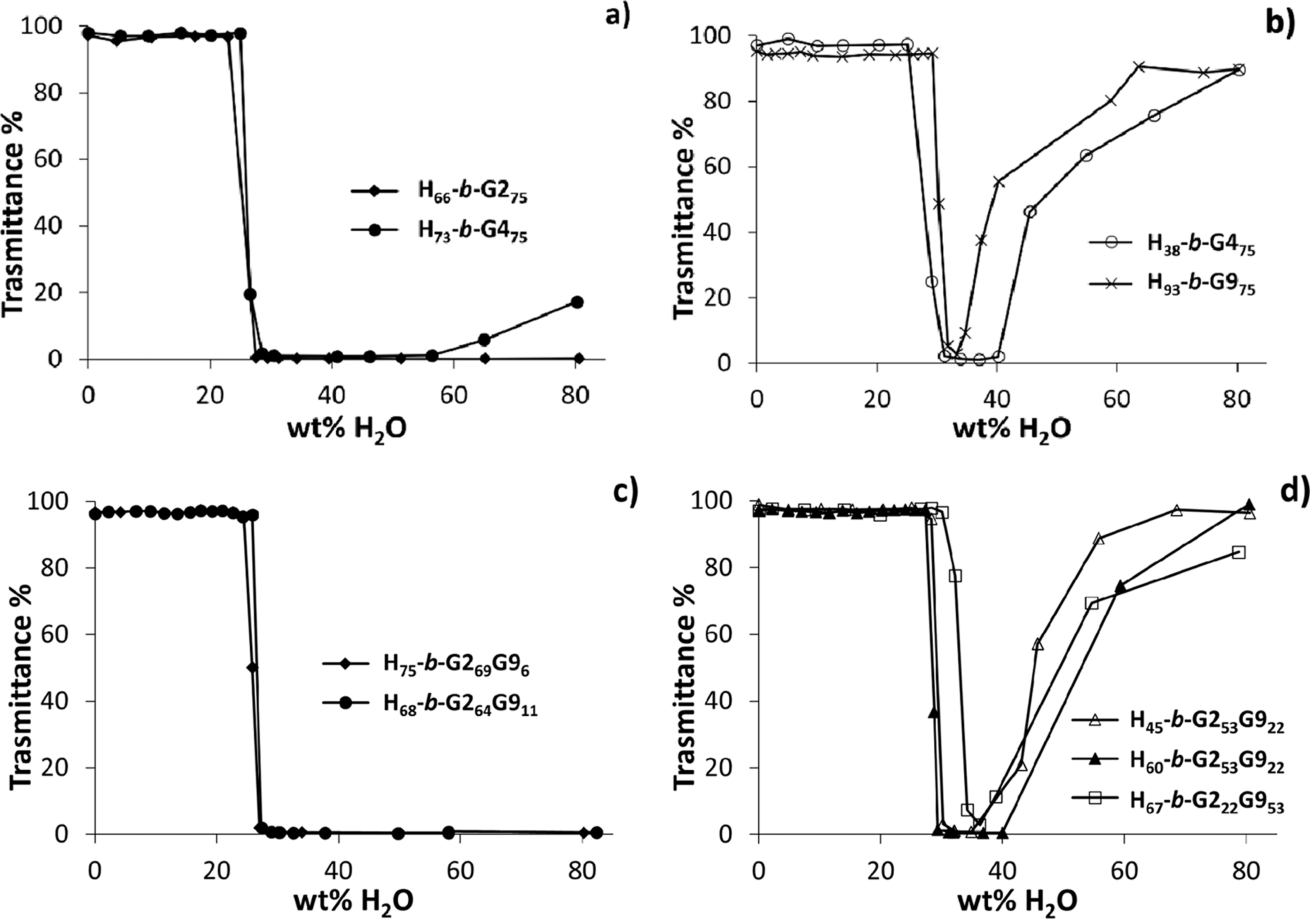
Transmittance recorded at λ = 700 nm, as a function of the weight percentage of water added to the DMF solution of block copolymers. Initial polymer concentration in DMF: 10 mg mL^–1^. (a) **H**_**q**_**-*b*-GX**_***m***_ copolymers exhibiting turbidity at high wt % water; (b) **H**_**q**_**-*b*-GX**_***m***_ copolymers exhibiting turbidity upon water addition, followed by transmittance increase at high wt % water; (c) **H**_**q**_**-*b*-G2**_***m***_**G9**_***n***_ copolymers exhibiting turbidity at high wt % water; (d) **H**_**q**_**-*b*-G2**_***m***_**G9**_***n***_ copolymers exhibiting turbidity upon water addition, followed by transmittance increase at high wt % water.

### 2.6 PIM Method

The polymers were first dissolved in deionized water in the presence of ∼1.5 equiv of NaOH relative to the total amount of acid units (HIABMA), which was calculated from the chemical structure of the polymer (*C*_polymer_ = 1.5 mg/mL, corresponding to [HIABMA] ≈ 10^–3^ M, depending on different lengths of HIABMA blocks). In most cases, after stirring for 30 min, the solutions became transparent and polymers were solubilized (as shown by DLS analysis). Measured pH values were in the 11–11.5 range. To solubilize **H**_**66**_**-*b*-G2**_**75**_ and **H**_**75**_**-*b*-G2**_**69**_**G9**_**6**_, it is necessary to reach pH 12.5.

The pH was then gradually lowered by the addition of 0.1 M HCl, and the formation of nanoaggregates was monitored by DLS.

### 2.7 Dynamic Light Scattering

DLS data were obtained with a Brookhaven Instruments Corp. BI-200SM goniometer equipped with a BI-9000AT digital correlator using a solid-state laser (125 mW, λ = 532 nm). Measurements of scattered light were made at a scattering angle θ of 90°. The nanoparticle formation by SD or PIM was monitored at 25 ± 0.1 °C. The experimental duration was in the range of 5–20 min, and each experiment was repeated two or more times. Cumulant analysis or CONTIN was used to fit the data.

Temperature-dependent experiments were carried out up to 70 °C, recording measurements every 2–5 °C, with an accuracy of ±0.1 °C. The samples were allowed to equilibrate for 20–45 min before each measurement until a stable diffuse intensity value was obtained. Solutions from the SD method were filtered with 0.1 μm filters, Durapore, prior to temperature-dependent experiments.

### 2.8 Titration Experiments

All titration experiments were conducted under an argon atmosphere at room temperature using the Crison Basic 20 pH meter. The samples were first dissolved in deionized water in the presence of molar excess of NaOH.

#### 2.8.1 Titration of HIABMA

The monomer HIABMA (3.0 mg, 0.014 mmol) was first dissolved in deionized water in the presence of ∼1.2 equiv of NaOH to obtain 2.9 mL of solution at *C* = 4.9 × 10^–3^ M.

The solution was then titrated at room temperature with 0.1 M HCl at a constant rate of 0.01 mL min^–1^.

#### 2.8.2 Titration of **H**_**38**_**-*b*-G4**_**75**_

5 mg of **H**_**38**_**-*b*-G4**_**75**_ (*M*_*n*_ = 30.6 kDa) was first dissolved in water in the presence of ∼1.5 equiv of NaOH relative to the total amount of HIABMA units to obtain 3.3 mL of solution at *C*_poly*m*er_ = 1.5 mg mL^–1^ (corresponding to [HIABMA] = 1.9 × 10^–3^ M). The solution was then titrated at room temperature with 0.1 M HCl as described above.

### 2.9 Determination of the Dissocation Degree Using UV–Vis Spectrophotometry

5 mg of polymer (**H**_**38**_**-*b*-G4**_**75**_ or **H**_**75**_**-*b*-G2**_**69**_**G9**_**6**_) was solubilized in 3.3 mL of NaOH to obtain *C*_poly*m*er_ = 1.5 mg/mL (corresponding to [HIABMA] = 1.9 × 10^–3^ M and [HIABMA] = 3.5 × 10^–3^ M, respectively) at pH 13. The UV–vis spectra of solutions were monitored using an HP 8452A diode array spectrophotometer. 0.1 M HCl was then added, and the spectra and pH were recorded in triplicate at regular intervals along the acidification process. A quartz cuvette of 0.1 mm optical path was used for recording the spectra. The spectra reported in Figure 4b,d are corrected for dilution. Once the spectra were obtained, the dissociation degree α was calculated as described in the text, and its evolution as a function of pH is reported in Figure 4a,c.

### 2.10 Cloud Point by UV–Vis Spectrophotometry

Cloud points were measured by turbidimetry at λ = 700 nm on a JASCO V-530 UV–vis spectrophotometer equipped with a Peltier Jasco EHC-477T.

A weighed amount of **G2**_**75**_, **G4**_**75**_, **G9**_**75**_ homopolymer, or **G2**_***m***_**G9**_***n***_ copolymer was placed in a quartz cuvette (1 cm path length) and diluted to the desired concentration using Milli-Q-grade water. Final polymer concentrations were 0.75–1.05 mg mL^–1^. The solutions were allowed to equilibrate at room temperature for at least 30 min, and heated at 1 °C min^–1^ while the transmittance was monitored at 700 nm. Cloud points were determined as 90% transmittance at 700 nm. 1.5 mg mL^–1^ of nanoaggregate solutions from the PIM and SD methods was directly placed in a quartz cuvette (1 cm path length).

### 2.11 TEM Measurements

Measurements were carried out with a FEI Tecnai 12 G2 Twin (FEI Company, Hillsboro, OR, USA), operating at 120 kV and equipped with an electron energy filter (Gatan GIF energy image filter) and a slow-scan charge-coupled device camera (Gatan multiscan). 10 μL of 1.5 mg mL^–1^ solution was deposited onto a 400 mesh copper grid covered with a very thin (about 20 nm) amorphous carbon film. The excess of liquid was removed by placing the grid onto a piece of filter paper and left to dry in air at room temperature for a few minutes. 2% w/v phosphotungstic acid (PTA) buffered at pH 7.3 was used for negative staining.

### 2.12 SAXS Measurements

SAXS measurements were performed at SAXSLab Sapienza with a Xeuss 2.0 Q-Xoom system (Xenocs SA, Grenoble, France), equipped with a micro-focus Genix 3D X-ray source (λ = 0.1542 nm) and a two-dimensional Pilatus3 R 300 K detector, which can be placed at a variable distance from the sample (Dectris Ltd., Baden, Switzerland). The beam size was defined through the two-pinhole collimation system equipped with “scatterless” slits of 0.5 mm × 0.5 mm. Calibration of the scattering vector *q* range, where *q* = (4π sinθ)/λ, with 2θ being the scattering angle, was performed using silver behenate. Measurements with different sample–detector distances were performed so that the overall explored *q* region was 0.04 nm^–1^ < *q* < 12 nm^–1^. Samples were loaded into vacuum-tight quartz capillary cells with a thickness of 1.5 mm and measured in the instrument sample chamber at reduced pressure (∼0.2 mbar) in a thermostatted holder, set at 25 °C unless otherwise specified. The two-dimensional scattering patterns were subtracted for the “dark” counts, then masked, azimuthally averaged, and normalized for transmitted beam intensity, exposure time, and subtended solid angle per pixel, by using the FoxTrot software developed at SOLEIL. The one-dimensional intensity *versus q* profiles were then subtracted for the solvent and cell contributions and put in absolute scale units (cm^–1^) by dividing with the known thickness. The different angular ranges were merged using the SAXS utilities tool.^51^ Guinier fit analysis and the indirect Fourier transform to calculate the pair distance distribution functions were performed with the SAS Data Analysis tools of the ATSAS package.^52^ Attempts to describe the sample scattering according to analytical models were performed with the software packages SasView (SasView version 5.0.2, http://www.sasview.org/,accessed September 7, 2019) and SASfit.^53^

### 2.13 NMR Spectroscopy

^1^H analyses were performed on a Bruker AVANCE II operating at a proton frequency of 300 MHz. All polymers were dissolved in CDCl_3_ or DMSO-*d*_6_ for NMR analysis. Chemical shifts were referred to the solvent signal and expressed in parts per million (δ scale).

#### 2.13.1 Determination of Monomer Conversion

An aliquot of the polymerization crude was transferred to an NMR tube, and DMSO was removed under high vacuum. The residue was then diluted with CDCl_3_ and monomer conversions were calculated from ^1^H NMR spectra: HIABMA (synthesis of block copolymers) and OEGMA (synthesis of homopolymers and random copolymers) vinyl monomer signals were determined (6.12 and 6.08 ppm, respectively) at time = 0 and *t*. Signals were normalized to terminal OEGMA signal OCH_3_ (Supporting Information, **e**, 3H). Conversion was calculated as follows
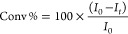


### 2.14 Gel Permeation Chromatography

GPC analyses were performed on a Hewlett Packard Series 1050 HPLC system equipped with a 1047A RI detector and a TSK gel alpha-4000 GPC column (Tosoh, Japan), using DMF with 0.1% (w/w) LiBr as the mobile phase at a flow rate of 0.8 mL min^–1^. The system was coupled to Clarity software version 6.2 (DataApex, Prague, The Czech Republic) for signal processing. Molecular weights are relative to monodisperse polyethylene oxide (PEO) standards (Agilent). The concentration of the polymeric solutions was ≈1–2 mg mL^–1^. Samples were filtered through poly(vinylidene difluoride) (PVDF) syringe filters 0.45 μm, 13 mm (Lab Service, Italy) prior to analysis.

### 2.15 High-Performance Liquid Chromatography

The purity of (HIABMA was assessed by HPLC analysis with UV detection (Agilent 1100 Series, λ = 230 nm). Column: Alltech Alltima C18, 5 μm, 250 mm × 2.1 mm. Solvent A: H_2_O/acetonitrile = 80:20, TFA 0.1%; Solvent B: acetonitrile/TFA 0.1%. Gradient from 100 to 20% A in 30 min. Samples were filtered through PVDF syringe filters 0.45 μm, 13 mm (Lab Service, Italy) prior to analysis.

## 3 Results and Discussion

### 3.1 Synthesis of Block Copolymers

We obtained several block copolymers of OEGMA (hydrophilic block) and HIABMA (hydrophobic block) as described in Scheme 1 via RAFT polymerization using CPDB as the chain-transfer agent. We used OEGMA with different pendent chain lengths (**G2**, **G4**, and **G9**), and the hydrophilic blocks were obtained either as **G2**, **G4**, or **G9** homopolymers or as **G2G9** random copolymers with different **G2**/**G9** ratios. Monomer conversions, as determined by ^1^H NMR, exceeded 95% in all cases, so we assumed degree of polymerization, DP = 75 (based on [*M*]_0_/[CTA] = 75:1, with [*M*]_0_ = initial monomer concentration and [CTA] = concentration of the chain-transfer agent in the reaction mixture) within experimental error. Therefore, we were able to extend the resulting macro-CTA (Scheme 1) by simply adding the HIABMA monomer to an aliquot of the reaction mixture and re-initiating the polymerization reaction, assuming that incorporation of the residual OEGMA in the second block would not affect our results significantly. Conversions for chain extension were in the 73 to >95% range (Table 1).

The composition of the block copolymers was confirmed by ^1^H NMR (Figures S1 and S2). The Đ of both macro-CTAs and block copolymers (GPC) was <1.2 (chromatograms: Figure S3). *M*_*n*_ values estimated by GPC are quite different from those calculated from ^1^H NMR conversion, owing to the different topology of POEGMA and their diblock copolymers with respect to the linear PEO standards used for calibration.^54^

### 3.2 Polymeric Micelles

None of the block copolymers in this study is soluble directly in water at neutral pH, and clear colloidal suspensions can only be obtained in most cases (see herein) by inducing self-assembly. To this end, we used two different strategies, that is, the SD and PIM methods. Results from both methods are summarized in Table 2.

**Table 2:** Summary of DLS Diameters (*D*_H_)^a^ Obtained for Aqueous Dispersions of Diblock Copolymer Micelles at 25 °C by SD^b^ and by PIM^c^

	*D*_H_ (nm)	
polymer	SD^b^	PIM^c^
**H**_**66**_**-*b*-G2**_**75**_	phase separation at H_2_O > 80%	N/A^d^
**H**_**38**_**-*b*-G4**_**75**_	39 ± 2	30 ± 2
**H**_**73**_**-*b*-G4**_**75**_	phase separation at H_2_O > 80%	40 ± 2
**H**_**93**_**-*b*-G9**_**75**_	34 ± 2	32 ± 2
**H**_**75**_**-*b*-G2**_**69**_**G9**_**6**_	phase separation	138 ± 2^e^
**H**_**68**_**-*b*-G2**_**64**_**G9**_**11**_	phase separation	49 ± 2
**H**_**45**_**-*b*-G2**_**53**_**G9**_**22**_	34 ± 2	29 ± 2
**H**_**60**_**-*b*-G2**_**53**_**G9**_**22**_	39 ± 2	26 ± 2
**H**_**67**_**-*b*-G2**_**22**_**G9**_**53**_	59 ± 2	24 ± 2

a Mean value of three measurements ± difference between maximum and minimum values.

b Polymers were dissolved in DMF and then water was slowly added to 80% (w/w %). Residual DMF was removed by dialysis. Final polymer concentration: 1.5 mg mL^–1^ in water.

c 1.5 mg mL^–1^ polymer in water with 1.5 equiv of NaOH, resulting in a measured pH 11–11.5. HCl (0.1 M) was then gradually added to reach pH 4.5–5.

d 1.5 mg mL^–1^ polymer in water at pH 12.4. After polymer addition, the measured pH was 12.3. HCl (0.1 M) was then gradually added and phase separation occurred at pH < 10.4.

e 1.5 mg mL^–1^ polymer in water at pH 12.6. After polymer addition, the measured pH was 12.5. HCl (0.1 M) was then gradually added to reach pH 4.

The SD method relies simply on the amphiphilicity of the copolymers to cause segregation of the uncharged PHIABMA block from the aqueous environment.^55^ We monitored the transmittance at λ = 700 nm as water was gradually added to the DMF polymer solution (Figure 1) and, when applicable, we determined the hydrodynamic diameter (*D*_H_) of nanoaggregates by DLS after dialysis (Table 2).

With 23–30% (w/w) added water, all block copolymer solutions exhibit turbidity (Figure 1), as polymer–polymer interactions dominate over polymer–solvent interactions (Figure 3, top). At this stage of self-assembly, large aggregates are formed and transmittance drops almost to zero. Upon further increasing the amount of water, some polymers (Figure 1b,d) show a nearly complete recovery of transmittance (*T* = 80–99%), while the others remain turbid up to 80 wt % of water (Figure 1a,c). As confirmed by DLS after dialysis (Table 2), only with some polymer compositions do large aggregates evolve into small micelles, causing an increase in transmittance, whereas phase separation occurs in all other cases. This different behavior seems to be correlated primarily to the hydrophilicity of the POEGMA block and secondarily to the PHIABMA/POEGMA block length ratio. The **G2** block in **H**_**66**_**-*b*-G2**_**75**_ is not hydrophilic enough to grant self-assembly into micelles (Figure 1a and Table 2), and the number of more hydrophilic **G9** units in the **G2G9** copolymer series needs to be relatively high to stabilize the nanoaggregates. In fact, **H**_**45**_**-*b*-G2**_**53**_**G9**_**22**_, **H**_**60**_**-*b*-G2**_**53**_**G9**_**22**_**,** and **H**_**67**_**-*b*-G2**_**22**_**G9**_**53**_ form micelles, whereas **H**_**75**_**-*b*-G2**_**69**_**G9**_**6**_ and **H**_**68**_**-*b*-G2**_**64**_**G9**_**11**_ phase-separate. Of the **G4** copolymers, only the one with the shortest hydrophobic block (**H**_**38**_**-*b*-G4**_**75**_) forms 39 nm micelles by SD (Table 2), thereby exhibiting an increase in transmittance as water exceeds 30% (Figure 1b), whereas **H**_**73**_**-*b*-G4**_**75**_ phase-separates. **H**_**93**_**-*b*-G9**_**75**_, which has the most hydrophilic POEGMA block, forms micelles of 34 nm in diameter, regardless of the high number of hydrophobic repeat units. It should be noted that all nanoaggregate solutions obtained with the SD method showed the presence of a minor population of larger nanoparticles (*D*_H_ = 200–400 nm, Figure S4). In Table 2, only the size of the smaller population is reported, since the larger one is not detected after filtration.

**Figure 2:**
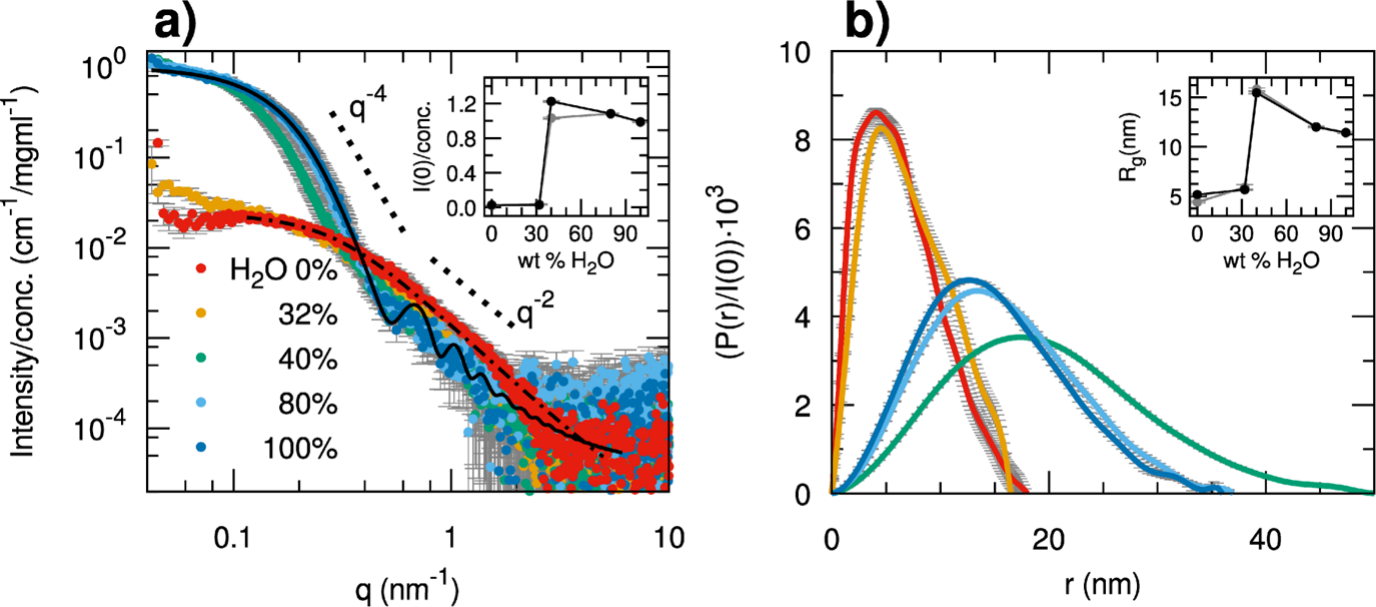
(a) SAXS data of **H**_**93**_**-*b*-G9**_**75**_ at different solvent compositions (water weight % in water–DMF mixtures) explored by the SD method for obtaining micellar aggregates. The data at 0% are fitted with the analytical model of a generalized Gaussian coil (dot-dashed black line)^56^ and the data at 100% water with the model of a block copolymer micelle (solid black line)^58^ whose parameters are reported in Table S3a,f, respectively; the characteristic slopes predicted for the Porod law (q-4) and for the Gaussian coil scattering (q-2) (the latter was also approximately followed by the micelle scattering profile at *q* > 0.5 nm^–1^) are visualized as dotted lines; in the inset, the values of the intensity extrapolated at zero angle normalized by the concentration *I*(0)/*c* are reported as a function of solvent composition. (b) Pair distance distribution functions *P*(*r*) obtained by indirect Fourier transform of the SAXS data in (a), normalized for the *I*(0) values; in the inset, the values of radius of gyration are reported as a function of solvent composition.

SAXS measurements (Figures 2 and S10) were performed at different solvent compositions across the SD preparation protocol for the copolymer **H**_**93**_**-*b*-G9**_**75**_, the one having the narrowest phase separation window (Figure 1b). The data show that the polymer is present in the form of a unimer when dissolved in DMF: in this condition, the scattering profile can be interpreted in terms of a generalized Gaussian coil^56^ having a radius of gyration (*R*_g_) of 5.5 nm, a Porod exponent of 2.08 (the excluded volume parameter is 0.48, slightly lower than the 0.5 expected for theta conditions of the polymer), and an intensity extrapolated at zero angle which is compatible with the nominal molecular weight calculated on the basis of the reported stoichiometry and the known dissolved concentration assuming a volume of 82 nm^3^ per polymer molecule, only 3% higher than the value estimated on the basis of the empirical method based on additivity^57^ (Tables S2, 3, Figure 2).

**Table 3:** Sizes and Calculated Molecular Weight at 25 °C as Inferred by SAXS Analysis of Polymeric Samples in a Unimer Form and in a Micellar Form Obtained by SD^b^ and by PIM^c^

	*c* (mg mL^–1^)	*R*_g_ (nm)	*D*_*m*ax_ (nm)	*I*(0) (cm^–1^)	MW^e^ (kDa)	*N*_agg_
**H**_**38**_**-*b*-G4**_**75**_						
**Unimer**^a^	1.5	2.71 ± 0.08	9 ± 1	0.020 ± 0.001	42 ± 1	1 ± 0.05
**SD**^b^	1.5	17.3 ± 0.4	60 ± 2	1.188 ± 0.025	2934 ± 63	101 ± 2.2
**PIM**^c^	1.5	8.64 ± 0.12	29 ± 2	0.566 ± 0.007	1398 ± 17	48 ± 0.6
**H**_**93**_**-*b*-G9**_**75**_						
**Unimer**^d^	2	4.4 ± 0.1	13 ± 1	0.043 ± 0.001	38 ± 1	1 ± 0.02
**Unimer**^d^	10	5.2 ± 0.1	18 ± 2	0.242 ± 0.003	44 ± 0.6	1 ± 0.01
**SD**^b^	1.5	11.4 ± 0.1	36 ± 2	1.48 ± 0.01	3510 ± 21	61 ± 0.4
**PIM**^c^	1.5	8.71 ± 0.08	28 ± 2	0.588 ± 0.006	1392 ± 13	24 ± 0.2
**H**_**60**_**-*b*-G2**_**53**_**G9**_**22**_						
**PIM**^c^	10	10.59 ± 0.02	32 ± 2	9.35 ± 0.02	3305 ± 8	97 ± 0.2

a Polymer solubilized in water with 1.5 equiv of NaOH, resulting in a measured pH of 11–11.5.

b Polymers were dissolved in DMF and then water was slowly added to 80% (w/w%). Residual DMF was removed by dialysis.

c Polymer was solubilized in water with 1.5 equiv of NaOH, resulting in a measured pH of 11–11.5. HCl (0.1 M) was then gradually added to reach pH 6.5–7.

d Polymer dissolved in DMF.

e .

Upon increasing the water content of the solvent, the SAXS data show a critical increase of the scattered intensity and size of inhomogeneities between 32 and 40%. At 40% water content, the profile corresponds to inhomogeneities with a maximum diameter of the order of 40–50 nm, whereas upon further adding water up to 80%, the aggregates slightly shrink to a maximum size of 35 nm and have a scattering profile substantially superimposable to that measured for the final dialyzed sample (100% water). By estimating the aggregation number from the absolute scattering intensity (Table 3), the final micelles obtained by the SD method should be composed of 61 ± 0.4 chains of **H**_**93**_**-*b*-G9**_**75**_. The scattering profile could be reasonably described by the form factor of a block copolymer micelle built as a homogeneous spherical core surrounded by a shell of Gaussian chains grafted on the surface.^58^ Initially, the molecular volumes and scattering length densities of the PHIABMA and POEGMA blocks (Table S1) were considered as fixed parameters for the core and shell compositions, respectively. However, to obtain a reasonable agreement in this case, it is necessary to account for a certain volume of water in the spherical core having a radius of 8.8 nm (43% volume fraction) and to also relax the scattering length density of the blocks in the shell to be slightly lower (Table S3f). This suggests that a model with full core–shell segregation having pure PHIABMA blocks in the core and pure POEGMA chains in the shell would not be fully compatible with the data collected for the micelles obtained by the SD method.

**Scheme 2:**
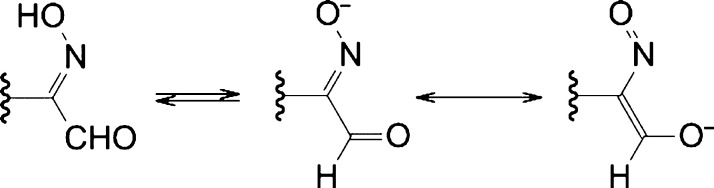
Dissociation of the 2-(Hydroxyimino)aldehyde Group and Charge Delocalization in the Anion

As an alternative to the SD technique, we exploited the acid–base properties of the HIA group (Scheme 2) to induce the formation of nanoaggregates.

**Figure 3:**
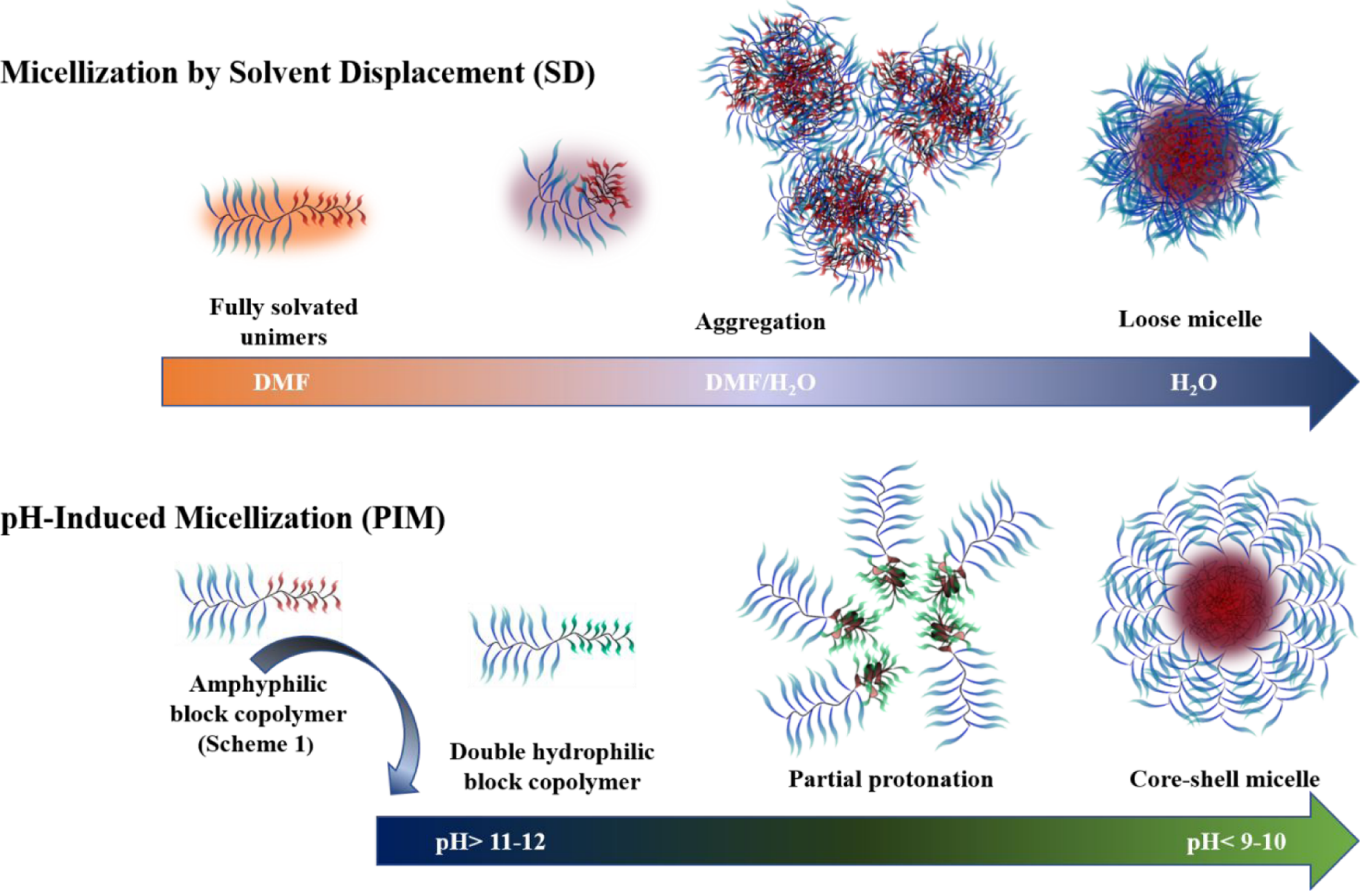
Schematic cartoon of micelle formation by SD and PIM techniques. Not drawn to scale. Blue ribbons: Hydrophilic PEG side chains; red ribbons and red areas: hydrophobic protonated HIA side chains; green ribbons: anionic oximate side chains; dark gray coils: polymethacrylate main chain.

To this end, we dissolved the polymers in excess NaOH_(aq)_ with respect to the HIABMA repeat units and then proceeded to lower the solution pH slowly to protonate the oxime residues. At high pH, the ionized PHIABMA block is a hydrophilic anionic polyelectrolyte, whereas, as the pH is lowered, the block is partly protonated and becomes progressively hydrophobic, thus inducing self-assembly (Figure 3, bottom). Nearly all copolymers are soluble in the presence of a 1.5 molar ratio of strong base with respect to the HIABMA units (polymer, 1.5 mg mL^–1^; resulting pH, 11.3–11.5). It is necessary to reach pH 12, instead, to solubilize **H**_**66**_**-*b*-G2**_**75**_ and **H**_**75**_**-*b*-G2**_**69**_**G9**_**6**_.

**Figure 4:**
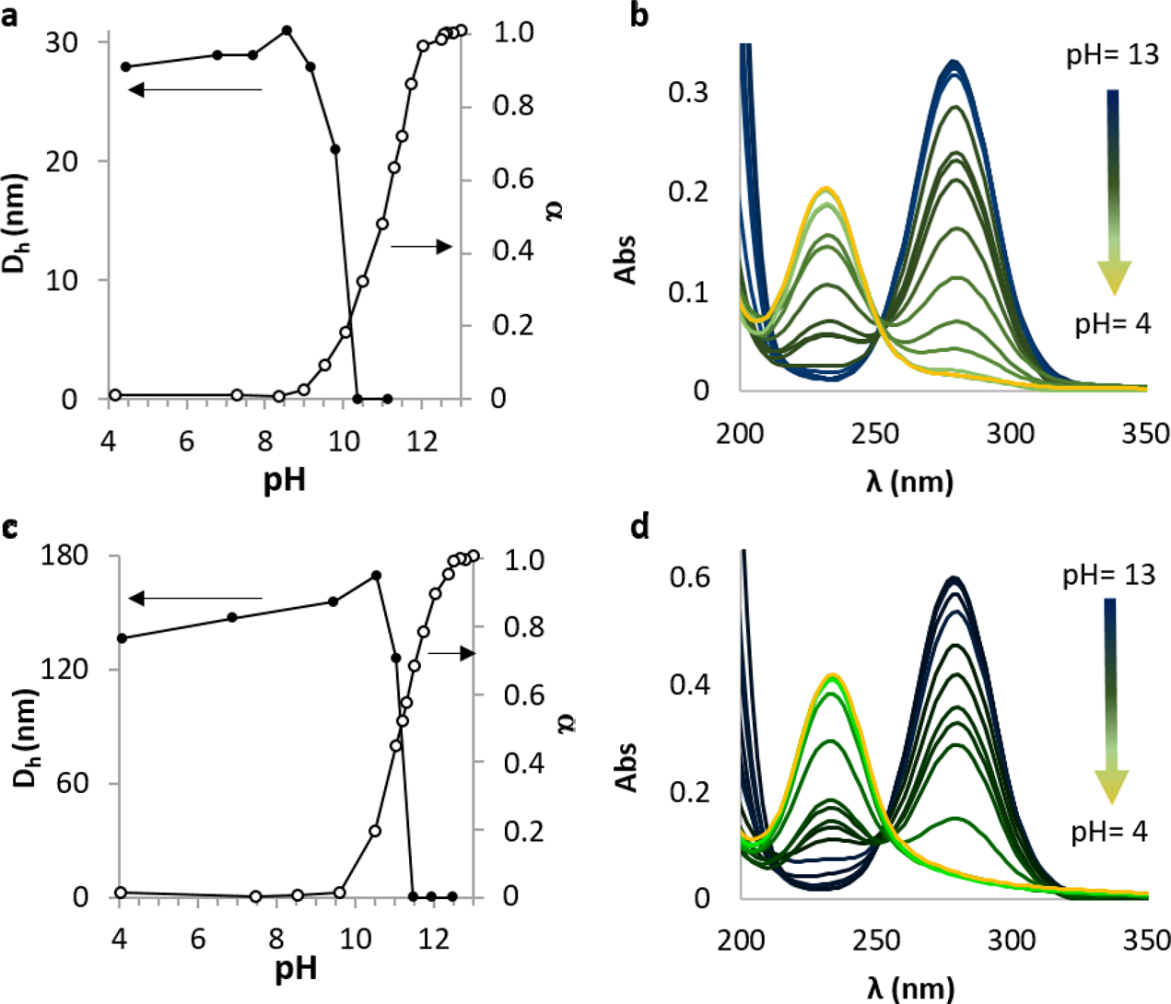
Micelle diameter (DLS, *D*_h_) and HIA degree of dissociation () as the pH is decreased from 11 or 12.5 to 4 by addition of 0.1 M HCl (initial polymer concentration, 1.5 mg mL^–1^): (a) **H**_**38**_**-*b*-G4**_**75**_ and (c) **H**_**75**_**-*b*-G2**_**69**_**G9**_**6**_. UV–vis spectra of (b) **H**_**38**_**-*b*-G4**_**75**_ and (d) **H**_**75**_**-*b*-G2**_**69**_**G9**_**6**_, 1.5 mg mL^–1^ in water at pH 13 to 4.0.

The titration curve of a **H**_**38**_**-*b*-G4**_**75**_ solution containing 1.5 molar excess of NaOH only exhibits one plateau, in contrast with that of HIABMA (Figures S5 and S6, respectively), in which two distinct plateaus are resolved. The titration curves of polyelectrolyte homopolymers, amphiphilic blocks, and random copolymers usually exhibit two distinct plateaus, one due to the neutralization of any excess strong base or acid and the second for the weak polyelectrolyte groups.^59,60^ However, if the polyelectrolyte is too weakly acidic or basic, its neutralization occurs at the same time as that of the strong base/acid, and the titration curve only exhibits one plateau. In order to assess the apparent dissociation degree α_app_ of the oxime groups as a function of pH, we solubilized **H**_**38**_**-*b*-G4**_**75**_ and **H**_**75**_**-*b*-G2**_**69**_**G9**_**6**_ in NaOH_(aq)_ at pH 13 to ensure α = 1, then we monitored the UV–vis spectra of solutions upon acidification (Figure 4b,d). HIA groups exhibit two bands at λ = 230 and 280 nm, corresponding to the undissociated form and to the conjugated base, respectively.^38^ At pH 13, only the 280 nm band is present and its intensity is the highest, whereas at pH 4 a residual absorbance at λ = 280 nm (*A*_280,*m*i*n*_) is also observed. We attribute (*A*_280,*m*ax_ – *A*_280,*m*i*n*_) to α_app_ = 1, and, at lower pH, α_app_ can be estimated as
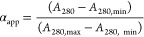
resulting in the α *versus* pH plot in Figure 4a,c. It is worth noting that, as could be expected, the polyacid block is overall a weaker acid than the monomer. In fact, based on the degree of dissociation α = 0.5, p*K*_a_ = 11.2–11.3 is estimated for the HIA groups in **H**_**38**_**-*b*-G4**_**75**_, whereas HIABMA has an estimated p*K*_a_ of 7.7–8.4 (see ref (38) and Figure S6). The effective p*K*_a_s of protonated polyelectrolyte repeat units can differ, in theory,^61^ by as much as 4 units from those of the monomers, although, in practice, differences are of the order of 1–3 units, also depending on experimental parameters, such as the concentration and presence of salts.^2,61^ Bütün et al.^28^ found p*K*_a_ values of 4.9 to 7.3 for the conjugated acid of a series of poly(2-dialkylaminoethyl methacrylates), that is 2–3 units lower than small-molecule analogues. On the other hand, the effective p*K*_a_ of 5 of the protonated pyridine units in poly(2-vinylpyridine) block copolymers with PEO^23^ is the same as the p*K*_a_ of the monomer,^62^ whereas Rodrigues et al.^63^ found, for the 4-vinylpyridinium units, p*K*_a_ = 4.64 in poly(4-vinylpyridine) (P4VP) and p*K*_a_ = 3.36–3.79 in different mPEG-*b*-P4VP block copolymers. It should be noted that not only are these values lower by at least 1 unit relative to the p*K*_a_ of the monomer (p*K*_a_ = 5.6 for 4-vinylpyridine),^62^ but this difference also depends on block copolymer composition. The dissociation constant of poly(acrylic acid) (p*K*_a_ = 4.26–5.25)^13,15,64,65^ is quite close to that of the corresponding monomer (p*K*_a_ = 4.25).^66^ On the other hand, poly(methacrylic acid) exhibits a distinct behavior, mostly related to hydrophobic interactions, that significantly affects its dissociation constant as a function of tacticity, changes in chain conformation at different ionization fractions, and the nature of counterions.^65,67^ The poly(4-vinylbenzoic) block in the zwitterionic copolymers by Liu and Armes^68^ has a p*K*_a_ of 7.1, that is about 3 p*K*_a_ units higher than the 4-vinylbenzoic acid. Compared to these examples, the PHIABMA-*b*-POEGMA copolymers in this study exhibit a much weaker acidity, thus providing pH responsivity in a totally different pH range (see herein).

**Figure 5:**
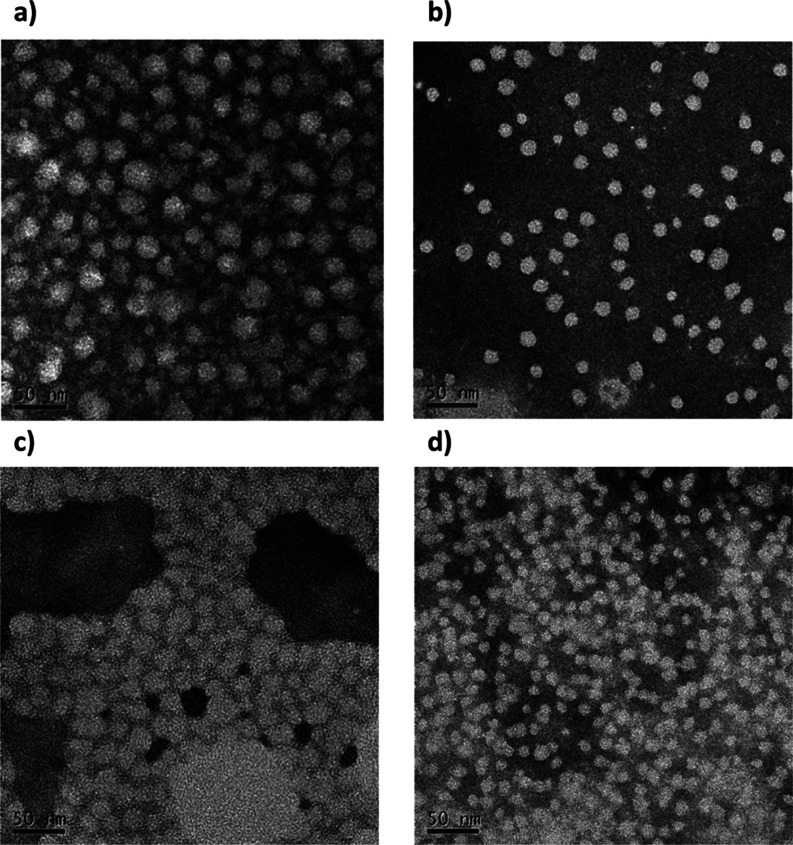
Negatively stained TEM micrographs of micelles of **H**_**38**_**-b-G4**_**75**_ obtained by (a) SD technique and (b) PIM; **H**_**93**_**-b-G9**_**75**_ obtained by (c) SD technique and (d) PIM. Deposition of polymer solutions, 0.5 mg mL^–1^ in neutral water, stained with 2% w/v PTA.

DLS analysis of the **H**_**38**_**-*b*-G4**_**75**_ and **H**_**75**_**-*b*-G2**_**69**_**G9**_**6**_ solutions (Figure 4a,c) shows that at pH > 11 the polymer chains are solubilized as unimers, whereas micelles form at a lower pH. The close correlation of the *D*_h_ and α curves in Figure 4a,c clearly shows that the increase in micelle diameter follows the pH-dependence of the degree of dissociation of HIA. Interestingly, **H**_**38**_**-*b*-G4**_**75**_ begins aggregating when 80% of the HIA groups are protonated (Figure 4a, α = 0.2), whereas **H**_**75**_**-*b*-G2**_**69**_**G9**_**6**_ aggregates already at 50% protonation. The PIM procedure was applied to all the polymers that we were able to solubilize in alkaline solution. With the only exception of **H**_**75**_**-*b*-G2**_**69**_**G9**_**6**_, the size of micelles obtained by PIM (Table 2; *D*_h_ = 24–49 nm) is compatible with a core–shell nanostructure. On the other hand, the aggregates resulting from **H**_**75**_**-*b*-G2**_**69**_**G9**_**6**_ (*D*_h_ = 138) should rather be thought of as *loose micelles.* In fact, the fully extended copolymer chain length of 150 vinyl repeat units, each of 0.25 nm *contour length*, would result in a core–shell micelle diameter of up to 75 nm, about half the observed value. It is worth noting that the relatively small amount of **G9** is just enough to stabilize large aggregates of an overall hydrophobic polymer, as recently observed with thermally induced nanoaggregates of POEGMA copolymers.^69^ Consistently, acidification of **H**_**66**_**-*b*-G2**_**75**_ alkaline solution resulted in outright phase separation due to lack of stabilizing **G9** units. PIM of all polymers is reversible, as determined by running a further alkalinization/acidification cycle. The micellar solutions were first adjusted to pH 7, and particle sizes were unchanged. Upon further alkalinization up to pH 11.5, the micelles disassembled, which then formed again with the same *D*_h_ upon acidification to pH 5. Micelles at pH 7 were unaltered upon storage at 4 °C for 24 h and 1 month. It is worth noting that **H**_**73**_**-*b*-G4**_**75**_ and **H**_**68**_**-b-G2**_**64**_**G9**_**11**_, which failed to yield micelles by the SD technique, formed, respectively, 40 and 50 nm aggregates by PIM. Furthermore, comparing the *D*_h_ of the micelles obtained with the two methods (Table 2), the ones induced by PIM are slightly but consistently smaller. The nanoparticles of **H**_**38**_**-*b*-G4**_**75**_ and **H**_**93**_**-*b*-G9**_**75**_ obtained with SD and PIM methods were observed by TEM using phosphotungstic acid for negative staining (Figure 5).

**Figure 6:**
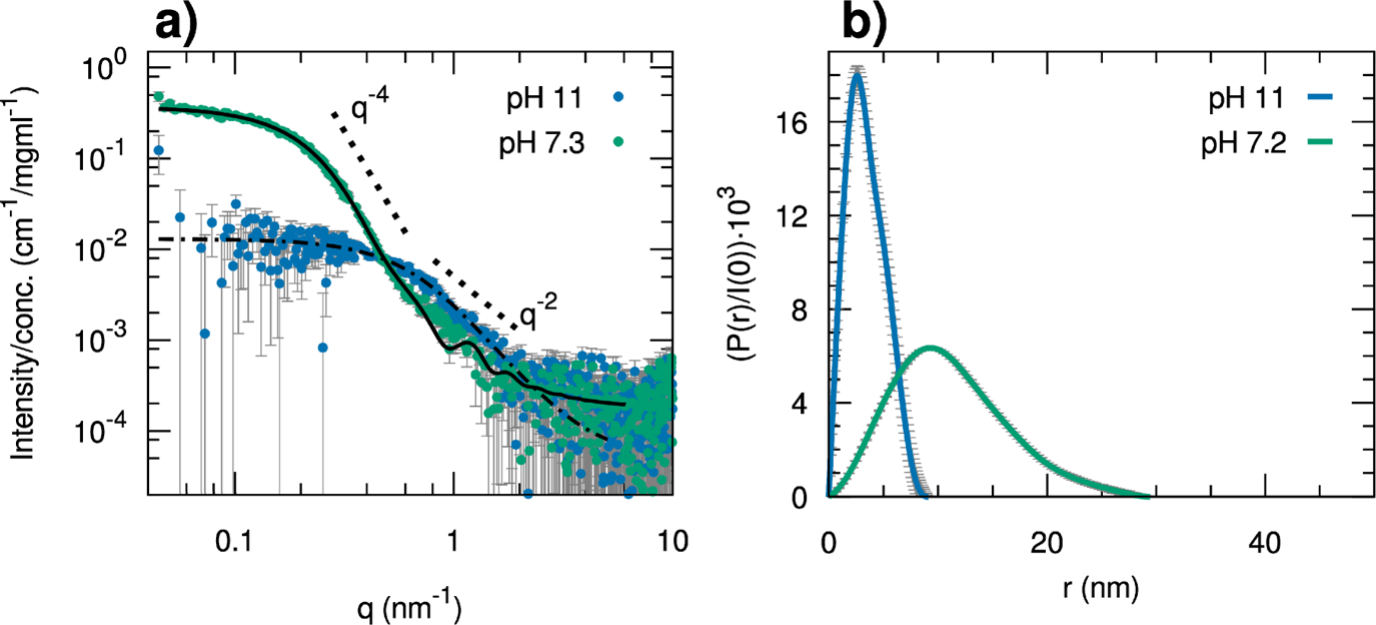
SAXS data of H_38_-*b*-G4_75_ dissolved at pH > 11 (blue dots) and after neutralization (pH 7.3, green dots), showing the formation of micelles according to the PIM approach. The data at pH 11 are fitted with the analytical model of a generalized Gaussian coil (dot-dashed black line)^56^ and the data at pH 7.3 with the model of a block copolymer micelle (solid black line),^70^ whose parameters are reported in Table S3c,d, respectively; the characteristic slopes predicted for the Porod law (*q*^–4^) and for the Gaussian coil scattering (*q*^–2^) are visualized as dotted lines; (b) pair distance distribution functions *P*(*r*) obtained by indirect Fourier transform of the SAXS data in (a), normalized for the *I*(0) values.

The images confirm the presence of nanoparticles with morphology attributable to core–shell micelles, the ones obtained by PIM appearing less polydisperse and smaller than those obtained by the SD method. For **H**_**38**_**-*b*-G4**_**75**_, micelles of 33 and 24 nm diameters, respectively, were obtained by SD and PIM methods (Figure 5a,b), while for **H**_**93**_**-*b*-G9**_**75**_ the diameters were 33 and 29 nm, respectively (Figure 5c,d). These values are slightly lower than those obtained by DLS (Table 2). This may be due to the contraction of the drying nanoparticles that can occur during TEM sample preparation. SAXS analysis of **H**_**38**_**-*b*-G4**_**75**_, **H**_**93**_**-*b*-G9**_**75**_, and **H**_**60**_**-*b*-G2**_**53**_**G9**_**22**_ copolymers on an absolute scale allowed us to further verify the structure of the aggregates directly in solution and to estimate their aggregation number (Table 3). For **H**_**38**_**-*b*-G4**_**75**_, the SAXS data collected after solubilization at pH > 11 (Figure 6) are in agreement with the presence of unimers, describable as coils with *R*_g_ of 2.7 nm, a Porod exponent of 2.86 (the excluded volume parameter is 0.35, suggesting that the copolymer is in a partially globular state in condition of not-so-good solvent) and an intensity extrapolated at zero angle which is compatible with the nominal molecular weight and the known dissolved concentration, assuming a volume of 39 nm^3^ per polymer molecule, only less than 3% smaller than the estimated value (Table S3c). After bringing the sample to neutral pH, micelles were obtained with an estimated aggregation number of 48 ± 0.6 and a diameter of the order of 30 nm (Figure 6).

**Figure 7:**
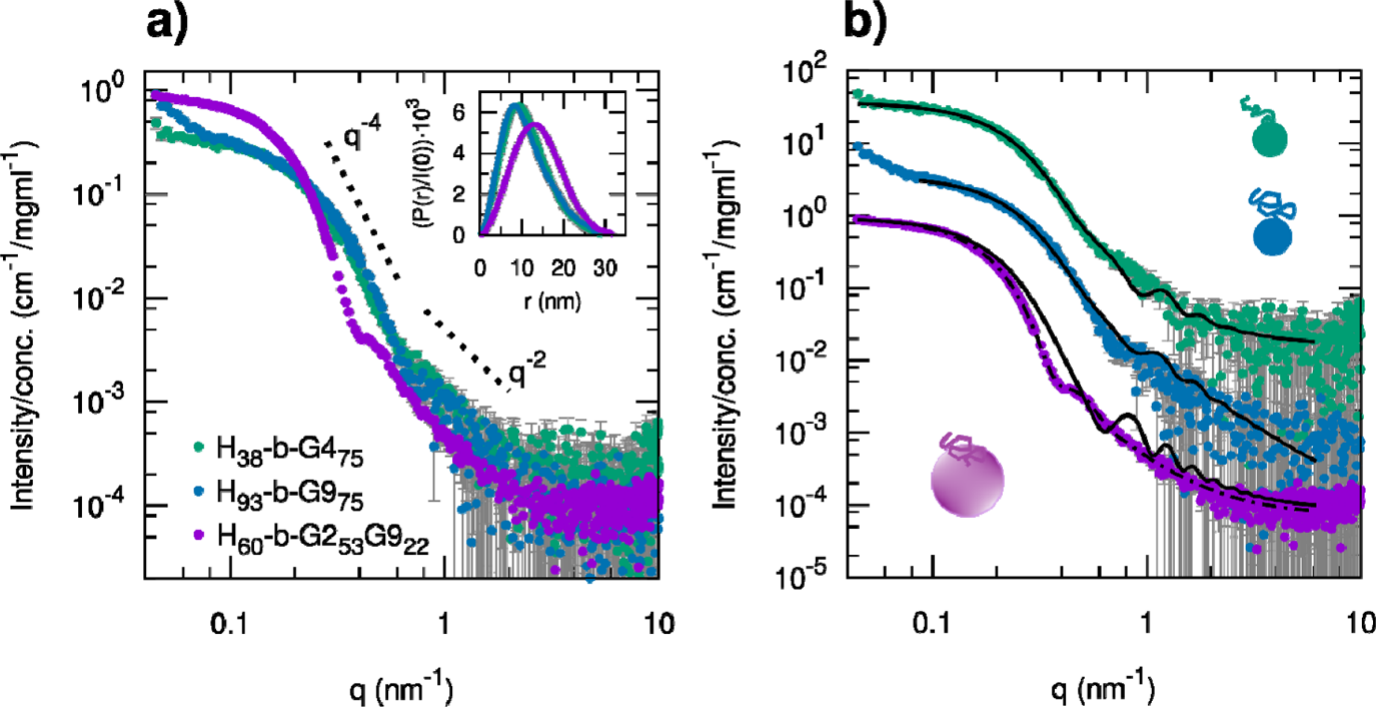
(a) Comparison of SAXS patterns recorded for three copolymers (**H**_**38**_**-*b*-G4**_**75**_, green; **H**_**93**_**-*b*-G9**_**75**_, blue; **H**_**60**_**-*b*-G2**_**53**_**G9**_**22**_, purple) showing the formation of micelles according to the PIM approach. In the inset, the corresponding pair distance distribution functions *P*(*r*) obtained by indirect Fourier transform and normalized for the *I*(0) values are compared. (b) Theoretical scattering intensity obtained according to the model of a block copolymer micelle imposing the estimated aggregation number (Table 3) and the SLD and volumes of the pHIA (core) and pOEGMA (brushes in the shell) blocks (Table S1) are shown as solid black lines (parameters are reported in Table S3d,e,g). For the **H**_**60**_**-*b*-G2**_**53**_**G9**_**22**_ micelles, the theoretical scattering intensity obtained by assuming a more flexible model implying less strong compositional segregation is shown as a dashed-dotted black line (parameters are reported in Table S3h). The data in (b) are shifted vertically by multiplying by a suitable factor (**H**_**93**_**-*b*-G9**_**75**_ × 10 and **H**_**38**_**-*b*-G4**_**75**_ × 100) for better visualization.

The scattering profiles of the micellar samples obtained by PIM for both **H**_**38**_**-*b*-G4**_**75**_ and **H**_**93**_**-*b*-G9**_**75**_ could be reasonably described with the model of a block copolymer micelle having the estimated aggregation numbers listed in Table 3 and the volumes and scattering length densities of the core and shell blocks fixed according to the expected composition of the PHIABMA and POEGMA blocks, respectively (Tables S1 and S3d,e, Figure 7). The overall micellar sizes in the two cases were found to be rather similar, with the difference that a longer PHIABMA block for **H**_**93**_**-*b*-G9**_**75**_ resulted in a lower aggregation number (24) and consequently a lower degree of surface coverage by the hydrophilic chains **G9**, slightly less expanded compared to the **G4** chains in the **H**_**38**_**-*b*-G4**_**75**_ micelles. In the case of **H**_**60**_**-*b*-G2**_**53**_**G9**_**22**_, such a model based on net core–shell segregation and a fixed aggregation number was not able to reproduce in an acceptable way the observed scattering profile and predicted too small aggregates (Figure 7b and Table S3g). A better description was possible by assuming a more flexible model accounting for a partial polydispersity of the core radius and different compositions of the core and shell, with a larger volume and a lower scattering length density for the block included in the core, possibly representing the inclusion of part of the mixed POEGMA blocks into the core, especially the more hydrophobic G2 units (Figure 7b and Table S3h), thus suggesting a less strong compositional segregation (see models in Figure 9).

The PIM and SD techniques are substantially different in that the former only affects the polyelectrolyte blocks, whereas in the SD technique, the solvation of both blocks is continuously affected as the H_2_O/DMF ratio is increased (Figure 3). It is reasonable to assume that, in SD, at the early stage of association by water addition (Figure 1; 23–30 wt % of water), the segregation of PHIABMA segments is not selective and that POEGMA segments are also included in the large nanoparticle hydrophobic cores (Figure 3, top).^71^ As the amount of water increases, kinetic trapping could limit or prevent, in some cases, the reorganization of the aggregates into core–shell micelles. Regarding PIM, aggregation starts occurring when a fraction of oxime groups is still dissociated (Figures 3 and 4), and it may be argued that the hydrogen bonding between oximate and oxime groups drives micellar core segregation along with hydrophobic interactions, thus resulting in more compact and orderly nanoaggregates. These differences in the nanoaggregation processes may explain the slightly smaller sizes of PIM micelles relative to those obtained with the SD technique. We do not have any dynamic data to assess whether the SD and/or PIM aggregates, which have kinetically driven morphologies, would be able to reach thermodynamic equilibration at room temperature over time or just stay frozen in a non-equilibrium state. However, it is reasonable to surmise that core–shell micelles are more similar to optimum morphology than loose ones. Even so, a clean correlation between block lengths and micelle size cannot be inferred from the data in Table 2. In the **H**_**38**_**-*b*-G4**_**75**_/**H**_**73**_**-*b*-G4**_**75**_ pair, the size of the PIM micelles increases from 30 to 40 nm as the hydrophobic block is increased from 38 to 73 repeat units. In the **H**_**45**_**-*b*-G2**_**53**_**G9**_**22**_/**H**_**60**_**-*b*-G2**_**53**_**G9**_**22**_ pair, which has a random copolymer as the hydrophilic block, the increase of the hydrophobic units (from 45 to 60) does not result in a significant difference in either SD or PIM micelle sizes. The fact that the two pairs behave differently does not come unexpected, as SAXS data of the PIM micelles formed by **H**_**38**_**-*b*-G4**_**75**_ and **H**_**60**_**-*b*-G2**_**53**_**G9**_**22**_ are best described with different models. In order to assess whether a clean correlation exists between block lengths and micelle sizes, a numerous set of systematically designed Hq-b-GXm block copolymers should be obtained and studied in a dedicated investigation.

### 3.3 Thermoresponsive Properties: Cloud Points and DLS

**Figure 8:**
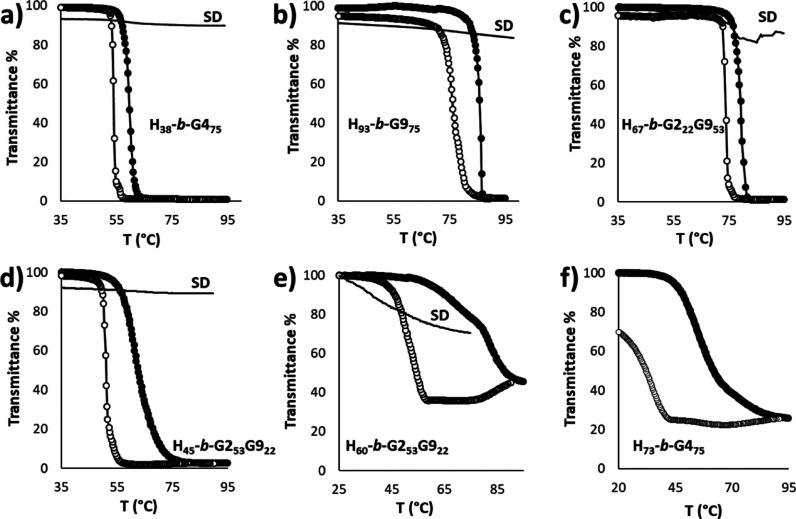
Turbidimetric measurements on 1.5 mg mL^–1^ polymeric micelles obtained by PIM. Heating run: full circles; cooling run: empty circles. Measurements on micelles obtained by SD (dashed line) are also plotted for comparison. pH = 7; λ = 700 nm; heating/cooling rate of 1 °C min^–1^. (a) **H**_**38**_**-*b*-G4**_**75**_; (b) **H**_**93**_**-*b*-G9**_**75**_; (c) **H**_**67**_**-*b*-G2**_**22**_**G9**_**53**_; (d) **H**_**45**_**-*b*-G2**_**53**_**G9**_**22**_; (e) **H**_**60**_**-*b*-G2**_**53**_**G9**_**22**_; (f) **H**_**73**_**-*b*-G4**_**75**_.

The transmittance (λ = 700 nm) of polymeric nanoparticles in water (1.5 mg mL^–1^) was measured as a function of temperature (Figure 8) to determine their turbidimetric cloud-point temperatures (*T*_CP_ at 90% transmittance, Table 4). POEGMA precursors were also analyzed, for comparison, at the same weight concentrations as in the micellar solutions (*C* = 0.75–1.1 mg mL^–1^). The *T*_CP_ of POEGMAs that spans 25 °C to >98 °C (Figure S7) is always characterized by a sharp transition and is consistent with literature values.^72,73^ The thermal behavior of SD and PIM micelles from the same polymers is remarkably different. Micelles obtained via SD show no definite *T*_CP_ values, with transmittance >70–80% even approaching 90 °C (Figure 8a–e), that is, at temperatures exceeding the *T*_CP_ of the corresponding POEGMA homopolymer (Table 4). The turbidimetric profiles of PIM micelles in Figure 8 show two different types of behavior. In Figure 8a–d, we observe a 100% drop in transmittance in the heating curve and some or no hysteresis during the heating/cooling cycle. These are the polymers that have either a shorter hydrophobic block (**8a** and **8d**) or a predominance of **G9** in the hydrophilic block (**8b** and **8c**). DLS analysis of these polymer solutions (Figure S8) shows an increase in micelle diameters (up to 3-fold) below the *T*_CP_ due to clustering of partially dehydrated and more compact micelles.^74,75^ On the other hand, in Figure 8e,f, it can be seen that the transmittance never falls to zero, and a larger difference between the heating and cooling scan is observed.

**Table 4:** Cloud Points of **H**_**q**_**-*b*-GX**_***m***_ and **H**_**q**_**-*b*-G2**_***m***_**G9**_***n***_ PIM Micelles and of Their Corresponding POEGMA Precursors

PIM micelles	*T*_CP_ (°C)^a^	POEGMA	*T*_CP_ (°C)^b^
**H**_**38**_**-b-G4**_**75**_	57	**G4**_**75**_	69
**H**_**73**_**-b-G4**_**75**_	48		
**H**_**93**_**-b-G9**_**75**_	82	**G9**_**75**_	>98
**H**_**75**_**-b-G2**_**69**_**G9**_**6**_	na^c^	**G2**_**69**_**G9**_**6**_	39
**H**_**68**_**-b-G2**_**64**_**G9**_**11**_	na^c^	**G2**_**64**_**G9**_**11**_	48
**H**_**45**_**-b-G2**_**53**_**G9**_**22**_			
**H**_**60**_**-b-G2**_**53**_**G9**_**22**_	56 na^c^	**G2**_**53**_**G9**_**22**_	66
**H**_**67**_**-b-G2**_**22**_**G9**_**53**_	76	**G2**_**22**_**G9**_**53**_	87

a *T*_CP_ = temperature at 90% transmittance (λ = 700 nm), heating scan. Polymeric micelles in water (1.5 mg mL^–1^).

b POEGMA precursors in water at the same weight concentrations as in the micellar solutions (*C* = 0.75–1.1 mg mL^–1^).

c No well-defined transition in the heating scan (Figures 8 and S9).

**Figure 9:**
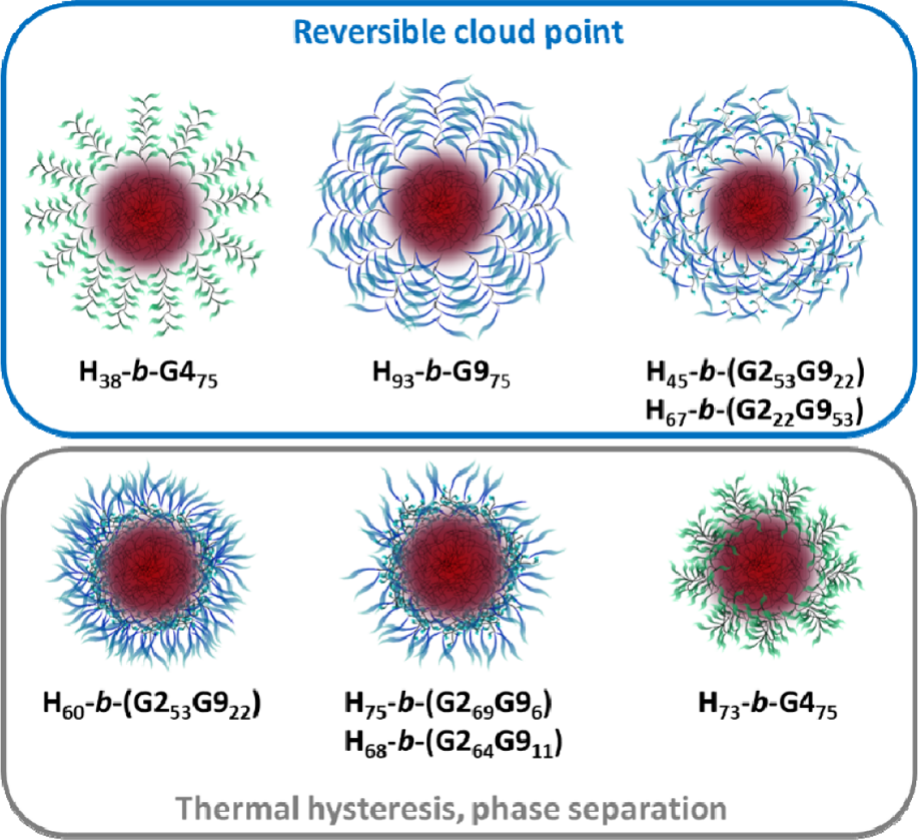
Schematic cartoon of different types of micelles obtained by PIM, as inferred from their thermal behavior and from the comparison among the SAXS analysis of **H**_**38**_**-*b*-G4**_**75**_, **H**_**93**_**-*b*-G9**_**75**_, and **H**_**60**_**-*b*-G2**_**53**_**G9**_**22**_.

These are the polymers that combine a longer hydrophobic block with hydrophilic segments containing either **G4** (Figure 8f *vs.***8a**) or **G2G9**, having less **G9** (Figure 8e *vs.***8c**) in the hydrophilic segment. We attribute this different thermal behavior to differences in the degree of compositional segregation within the micellar structure, as indicated by comparing the SAXS analysis of **H**_**60**_**-*b*-G2**_**53**_**G9**_**22**_*versus***H**_**38**_**-*b*-G4**_**75**_ and **H**_**93**_**-*b*-G9**_**75**_. A schematic representation of these differences is found in Figure 9. A peculiar thermal behavior has been described for **G2**_***n***_**GX**_***m***_ copolymers with *X* = 20–45 and *n* ≫ *m*.^69,75,76^ In these cases, a multistep thermal transition was observed due to the dehydration of the polymer backbone consisting of **G2**. The polymer chains adopt a compact globular structure induced by the intra- and intermolecular aggregation of the polymeric chains, whereas the segments with the longer EG chains remain hydrated.

As expected, *T*_CP_ values of the block copolymer PIM micelles (Table 4) are about 10–15 °C lower than those of their POEGMA precursors due to the close proximity of the polymeric chains in the shell and to the effect of the hydrophobic PHIABMA block. In fact, a drop of 10–20 °C in the *T*_CP_ of the POEGMA block has been observed in other examples of micelle-forming block copolymers.^77,78^**H**_**93**_**-*b*-G9**_**75**_ exhibits the most pronounced drop in *T*_CP_ relative to its **G9**_**75**_ precursor (82 °C *vs* >98 °C). We surmise that this is due to the presence of the longest hydrophobic block (93 repeat units) relative to the hydrophilic blocks. In fact, a sizable effect of the hydrophobic block length is also found comparing **H**_**38**_**-*b*-G4**_**75**_ and **H**_**73**_**-*b*-G4**_**75**_, which exhibit 12 and 21 °C drop in *T*_CP_, respectively, relative to **G4**_**75**_. Furthermore, the more hydrophobic **H**_**73**_**-*b*-G4**_**75**_ exhibits layered phase separation upon further heating, leading to an anomalous transmittance trace also in the cooling scan (Figure 8f). On the other hand, micelles that have very similar hydrophobic block length and the number of ethylene glycol units in the hydrophilic block exhibit similar *T*_CP_s (**H**_**38**_**-*b*-G4**_**75**_ and **H**_**45**_**-*b*-G2**_**53**_**G9**_**22**_, 57 and 56 °C, respectively, Figure 8a,d), in analogy with their corresponding precursors (**G4**_**75**_ and **G2**_**53**_**G9**_**22**_, 69 and 66 °C, respectively).

**Figure 10:**
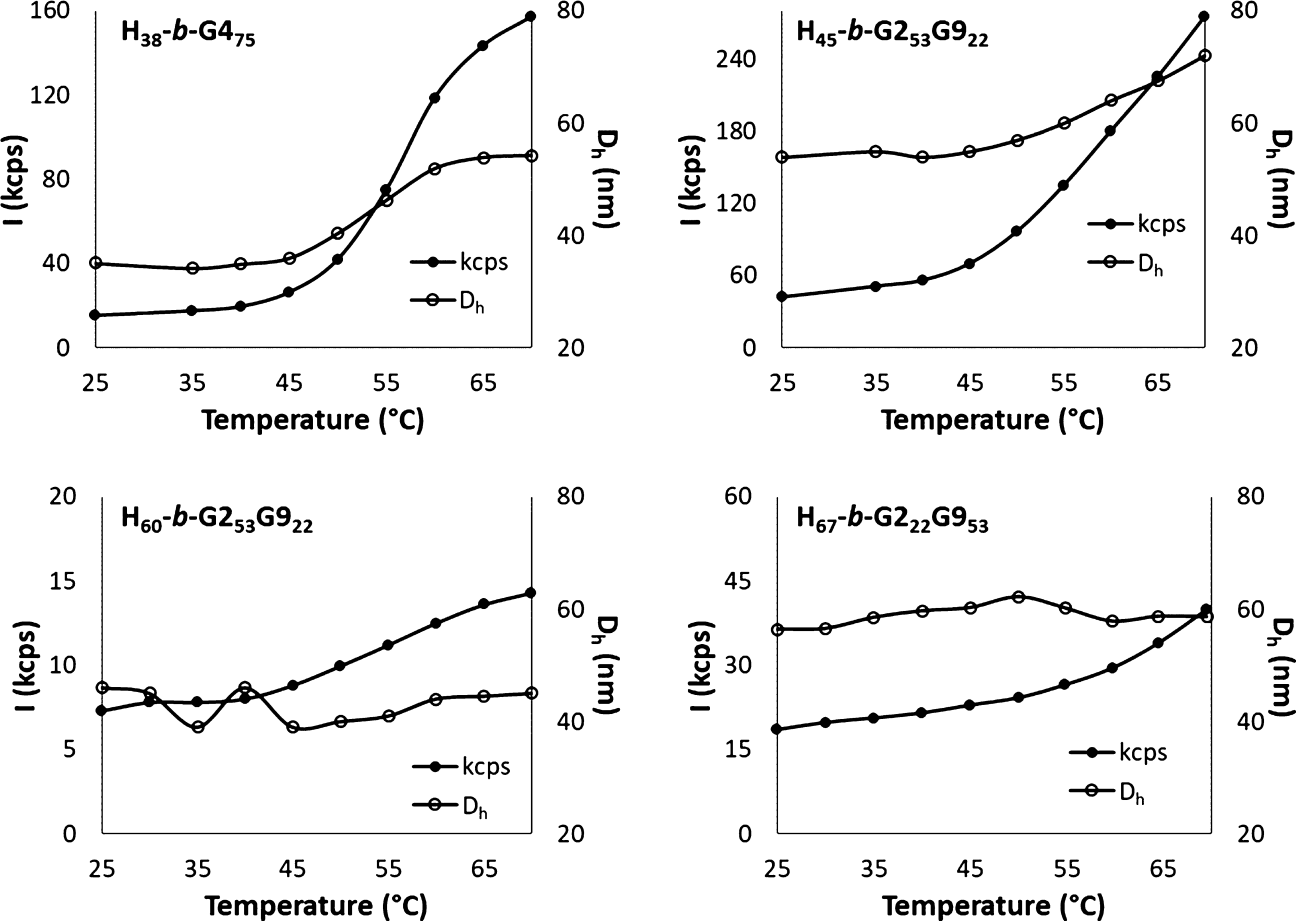
Scattered intensity and *D*_h_ (DLS) as a function of temperature of 1.5 mg mL^–1^ SD micelles in water.

Regarding **H**_**68**_**-*b*-G2**_**64**_**G9**_**11**_ and **H**_**75**_**-*b*-G2**_**69**_**G9**_**6**_, their thermal transitions (Figure S9) are again very broad and extend to temperatures that are not compatible with the composition of the **G2G9** copolymer. Furthermore, irreversible macroscopic precipitation occurs upon heating. It is possible that, due to the poor hydrophilicity of their **G2G9** chains, they do not assemble into well-defined core–shell micelles but rather as loose micelles with only some OEG_9_ chains on the outer surface. In fact, as discussed above, the size of **H**_**68**_**-*b*-G2**_**64**_**G9**_**11**_ nanoaggregates (*D*_h_ = 49), despite being compatible with a core–shell structure, is twice the size of all other small micelles. Nanoaggregates of **H**_**75**_**-*b*-G2**_**69**_**G9**_**6**_, instead, are too large (*D*_h_ = 138 nm) for a core–shell structure. DLS data obtained for micelles prepared by SD (Figure 10) allowed us to monitor temperature-induced aggregation up to 70 °C even in cases where no significant transmittance drop was observed by spectrophotometry.

Compared with the aggregation observed by DLS on PIM micelles (Figure S8), SD micelle aggregation is limited upon heating, with little or no *D*_h_ increase. Some differences in terms of scattering intensity are related to the balance between the hydrophilic and hydrophobic blocks. In fact, **H**_**45**_**-*b*-G2**_**53**_**G9**_**22**_ exhibits a significant increase in scattering intensity (42–257 kcps) along with an increase in aggregate diameter (from 54 to 72 nm), which is amenable to temperature-induced intermicellar aggregation. **H**_**38**_**-*b*-G4**_**75**_, which resembles **H**_**45**_**-*b*-G2**_**53**_**G9**_**22**_ in terms of length of hydrophobic block and overall number of EG units, also exhibits intermicellar aggregation, with an increase in both the scattering intensity and aggregate dimensions (kcps: 16 to 157; *D*_H_: 35–54 nm). On the other hand, no aggregation is induced by temperature on **H**_**60**_**-*b*-G2**_**53**_**G9**_**22**_ and **H**_**67**_**-*b*-G2**_**22**_**G9**_**53**_ (7–14 kcps and 19–40 kcps, respectively, with no change in *D*_H_). As we hypothesized for **H**_**60**_**-*b*-G2**_**53**_**G9**_**22**_ PIM micelles (see above), the SD method promotes the incorporation of the more hydrophobic portions of the POEGMA block (e.g., the backbone of the **G4** block or the **G2**-rich portions of the **G2G9** blocks) into the PHIABMA core (Figure 3). The copolymers that are rich in **G9** (i.e., **H**_**45**_**-*b*-G2**_**53**_**G9**_**22**_, **H**_**60**_**-*b*-G2**_**53**_**G9**_**22**_, and **H**_**67**_**-*b*- G2**_**22**_**G9**_**53**_) form micelles, either because the **G2**_***x***_**G9**_***y***_ blocks are all extended out of the PHIABMA core, or because the incorporation of **G2**-rich segments in the core leaves the corona enriched in hydrophilic **G9**. The smaller fraction of **G9** in **H**_**75**_**-*b*- G2**_**69**_**G9**_**6**_ and **H**_**68**_**-*b*- G2**_**64**_**G9**_**11**_, instead, would not be sufficient to provide a stabilizing hydrophilic shell, thereby resulting in phase separation. This could also explain why micelles obtained by SD are always larger than those prepared by PIM (Table 2). The slight increase in intensity at constant *D*_H_ observed for **H**_**60**_**-*b*-G2**_**53**_**G9**_**22**_ and **H**_**67**_**-*b*-G2**_**22**_**G9**_**53**_ could be linked to an increase in the refractive index of the nanoparticles due to partial dehydration of the micelle cores.

## 4 Conclusions

Using poly(HIABMA)-*b*-poly(OEGMA) copolymers as a case study, we have shown that PIM can promote the formation of monodisperse, stable, and reversible core–shell micelles from polymers that, through the SD technique, fail to form nanoaggregates or yield loose nanostructures. As a result, pH-induced micelles were thermoresponsive, whereas micelles obtained by SD lacked this useful property. Since the aggregation process of the core–shell micelles is governed by the pH-induced switch of the polyelectrolyte block from hydrophilic to hydrophobic, the thermal properties of the nanoaggregates are not only influenced by the composition of the hydrophilic corona but also by the length of the hydrophobic block. In fact, the right balance between hydrophobic and hydrophilic segments must be ensured to observe well-defined and reversible cloud points and to be able to tune the thermoresponsivity of polymeric micelles. PIMoffers a further advantage in that dilution is limited with respect to SD. In fact, with the pH-based technique, we were able to obtain micellar suspensions about 10 times more concentrated than by SD. The use of the weakly acidic, multi-responsive 2-(hydroxyimino)aldehyde group as an alternative to relatively stronger acids (acrylic or vinylbenzoic) provides the opportunity to combine the advantages of PIM with the formation of micelles that are stable at physiological pH while retaining the potential to respond to temperature and light. Including a weak acid (p*K*_a_ > 8) in the structure of hydrophobic segments in block copolymers may prove a general strategy for the fabrication of nanoaggregates through pH-gradient techniques.

## Supporting Information Available

NMR spectra, GPC chromatograms, DLS graphs, titration curves, turbidimetric measurements, and SAXS spectra (PDF)
