# Supplementary material for: Influence of Nanoaggregation Routes on the Structure and Thermal Behavior of Multiple-Stimuli-Responsive Micelles from Block Copolymers of Oligo(ethylene glycol) Methacrylate and the Weak Acid [2-(Hydroxyimino)aldehyde]butyl Methacrylate
Source: Langmuir. 2022 Nov 8;38(46):14371–86. doi: 10.1021/acs.langmuir.2c02515 (PMC9686140; doi:10.1021/acs.langmuir.2c02515)
Supplement: Supplementary file 1 — la2c02515_si_001.pdf [file la2c02515_si_001.pdf]

SUPPORTING INFORMATION

Influence of nanoaggregation routes on the structure and thermal behavior of multiple-stimuli responsive micelles from block copolymers of OEGMA and the weak acid [2-(hydroxyimino)aldehyde]butyl methacrylate

Irene Antignano, Francesca D'Acunzo*, Davide Arena, Stefano Casciardi, Alessandra Del Giudice, Francesca Gentile, Maria Pelosi, Giancarlo Masci*, Patrizia Gentili

Sommario

Figure S1. 2
Figure S2. 3
Figure S3. 4
Figure S4. 5
Figure S5. 5
Figure S6. 6
Figure S7. 6
Figure S8. 7
Figure S9. 7
Table S1 8
Table S2. 9
Table S3. 10
Figure S10. 17

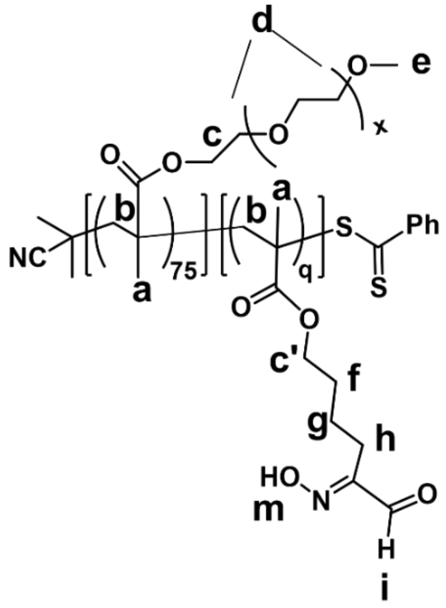

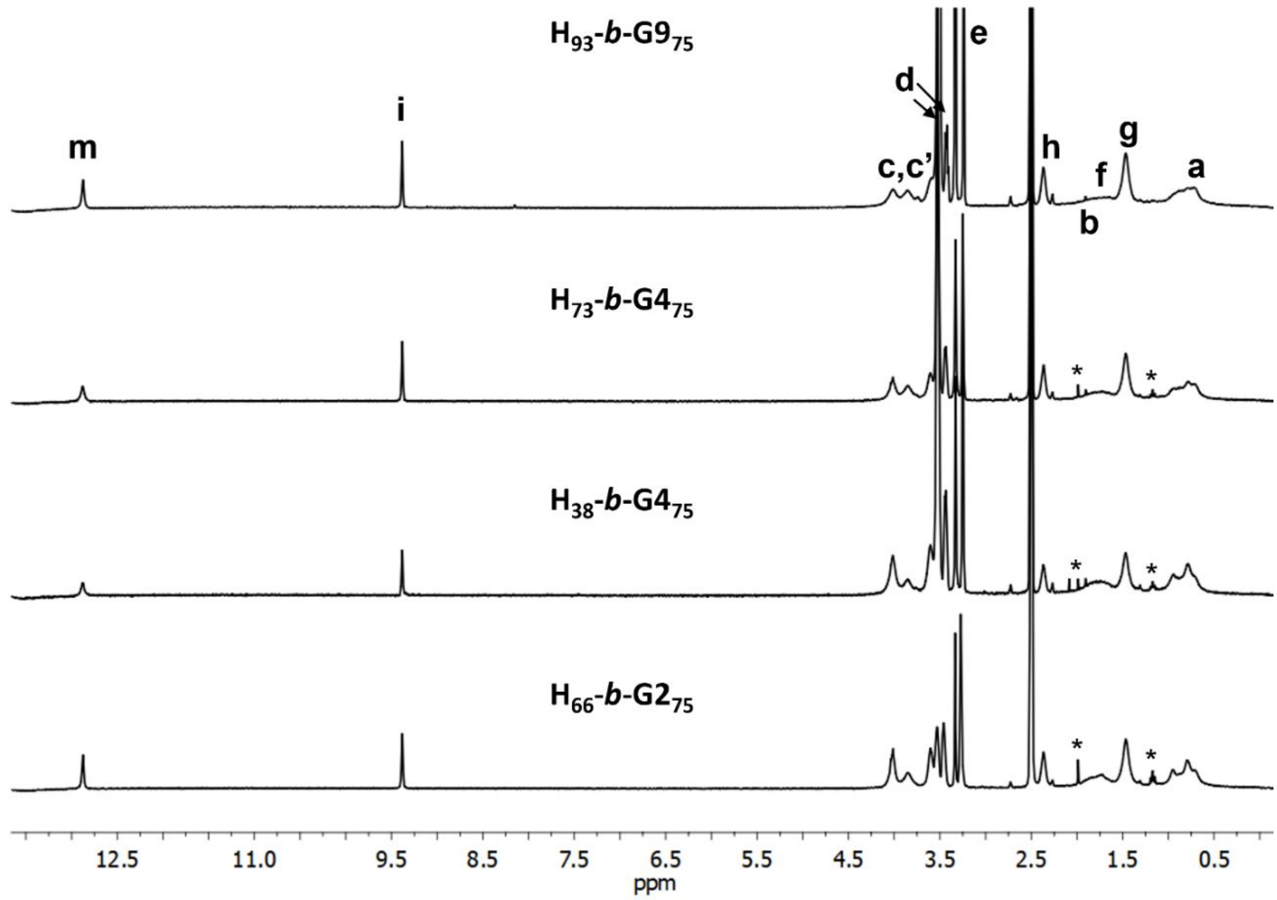

Figure S1. ^{1}H-NMR spectra of $\mathbf{H_q}$-***b*****-**$\mathbf{GX_m}$ (300 MHz, DMSO-d_6, δ). Diagnostic signals: 12.88 (s, **m**), 9.38 (s, **i**), 3.86 (br, **c'**), 2.37 (br, **h**), 1.47 (br, **g**).

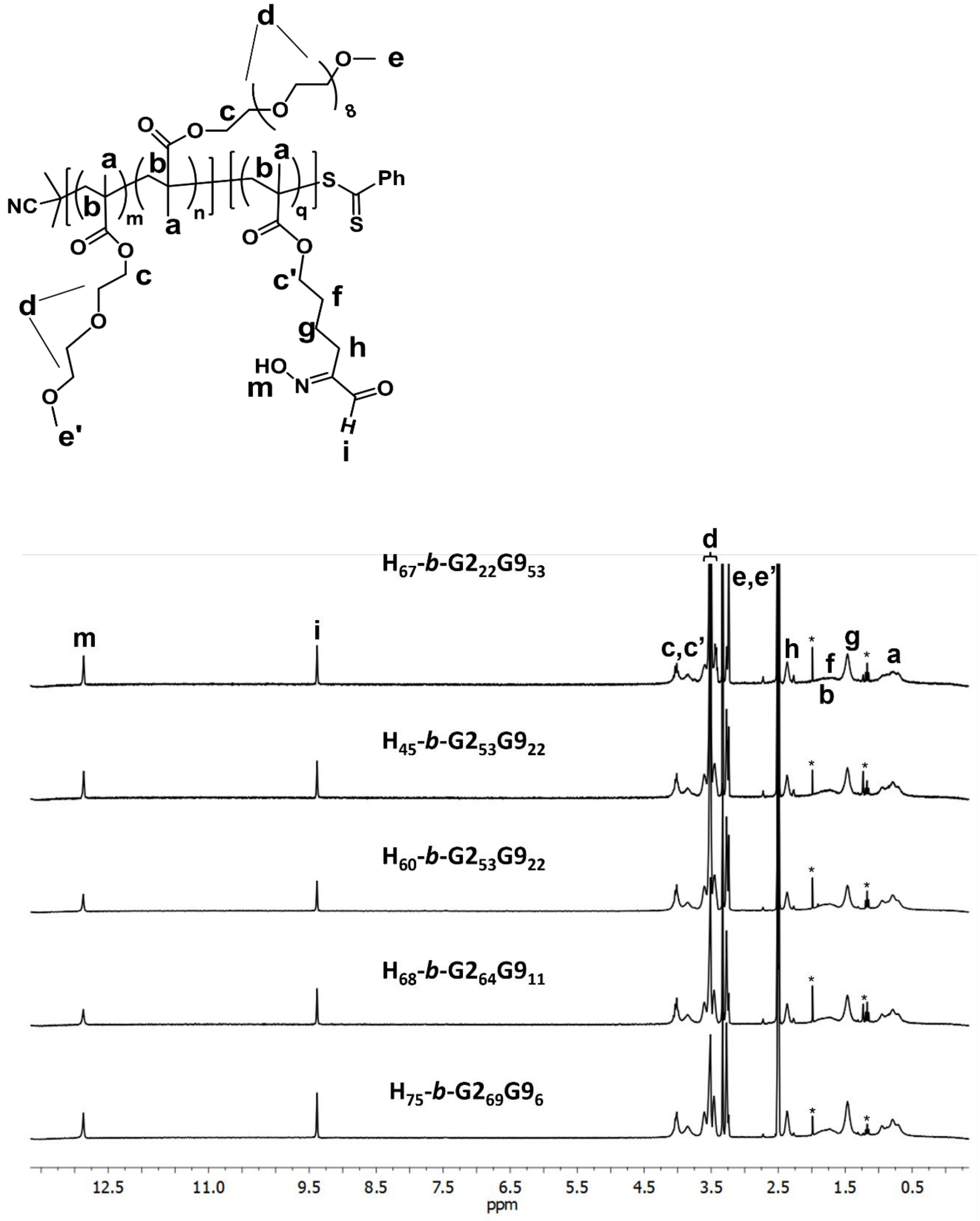

Figure S2. ^{1}H-NMR spectra of $\mathbf{H_q}$-***b*****-**$\mathbf{G2_mG9_n}$ (300 MHz, DMSO-*d*$_6$, δ). Diagnostic signals: 12.88 (s, **m**), 9.38 (s, **i**), 3.86 (br, **c’**), 2.37 (br, **h**), 1.47 (br, **g**).

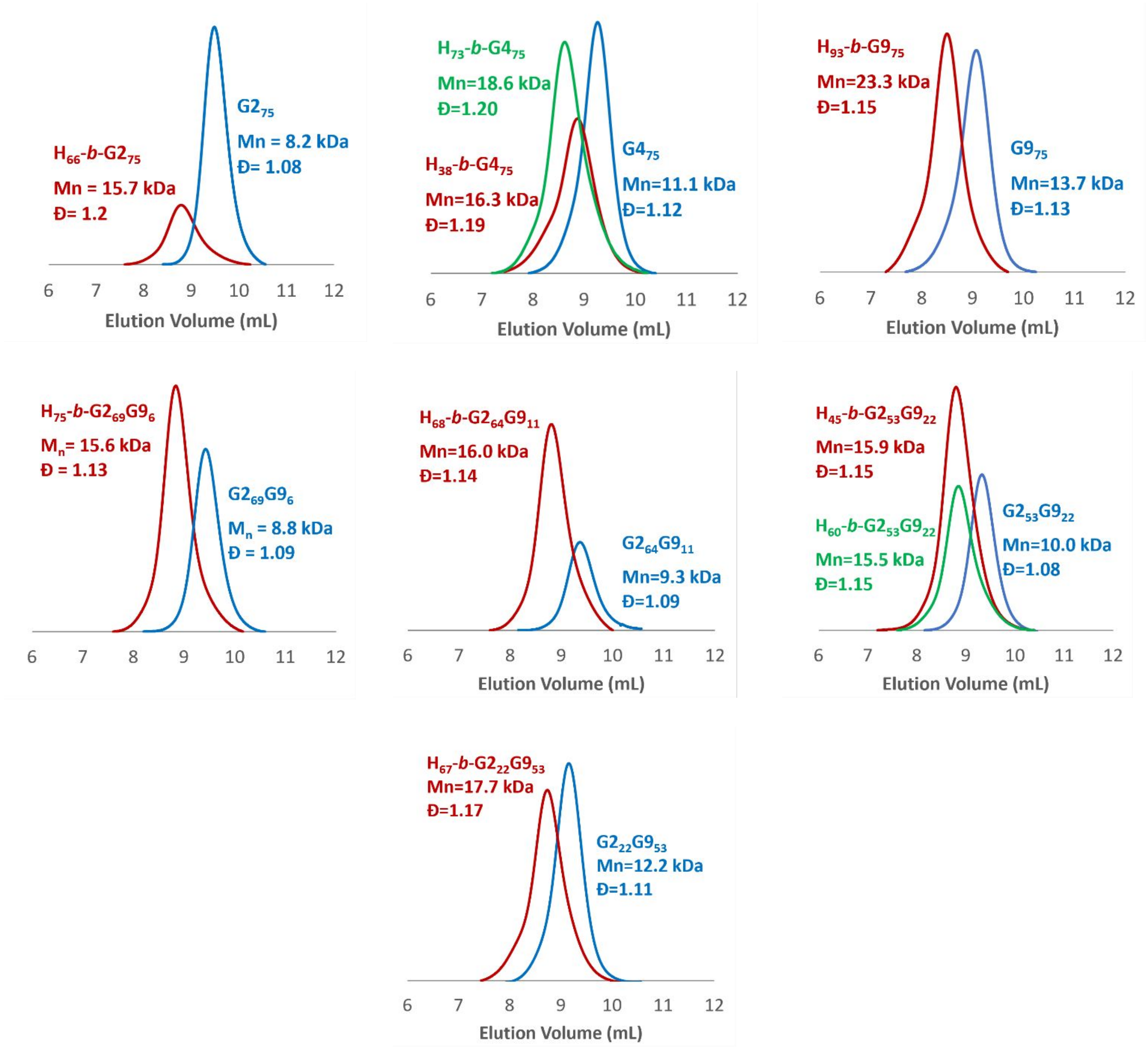

Figure S3. GPC traces (DMF/LiBr 0.1%; 0.8 mL min^{-1}) of $\mathbf{GX_m}$ and $\mathbf{G2_mG9_n}$ macro-CTAs (blue) and corresponding block copolymers (red and green) $\mathbf{H_q}$-***b***-$\mathbf{GX_m}$ or $\mathbf{H_q}$-***b***-$\mathbf{G2_mG9_n}$.

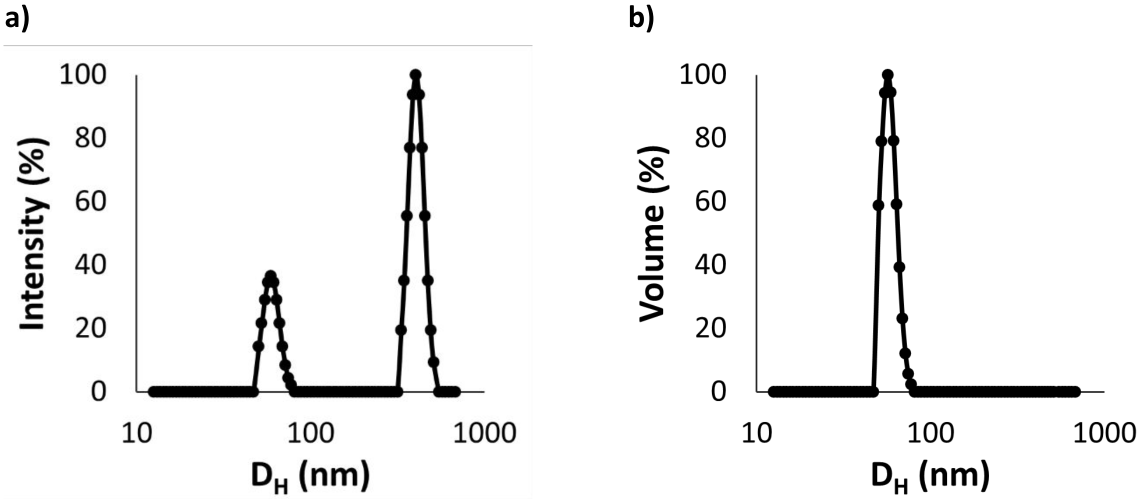

Figure S4. Intensity size distribution (a) and volume size distribution (b) measured by DLS at room temperature for micelles of $\mathbf{H_{67}}$-***b***-$\mathbf{G2_{22}G9_{53}}$ (1.5 mg mL^{-1} in water) obtained by SD method. The volume fraction of the larger-sized population is negligible.[1]

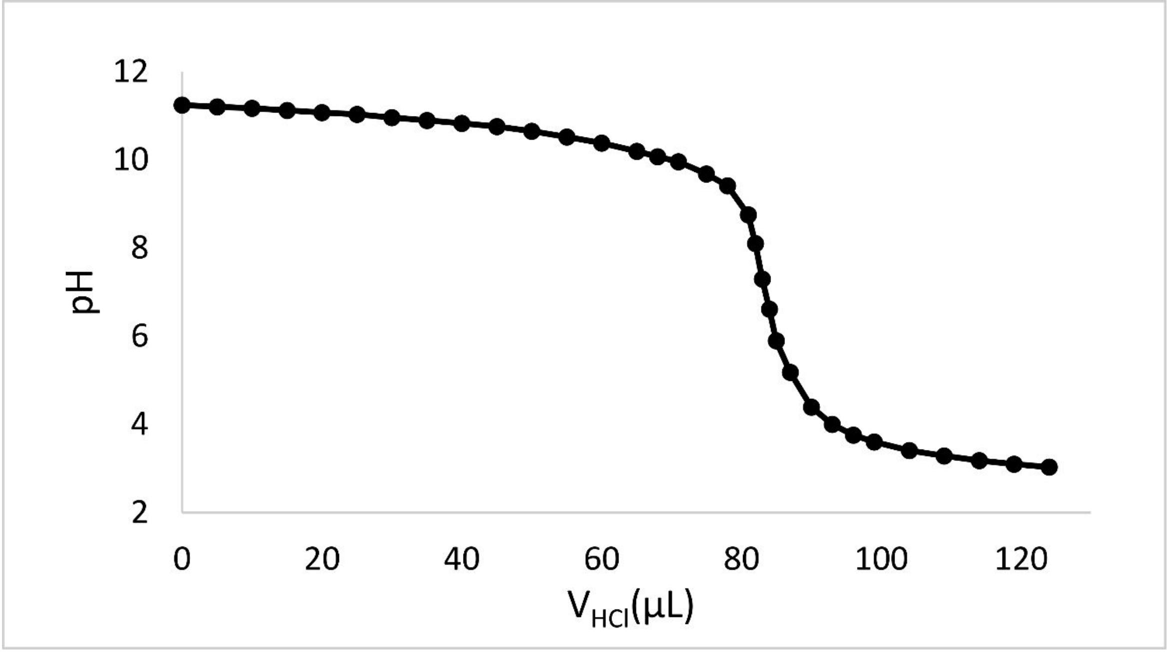

Figure S5. Titration curve of 5.0 mg of $\mathbf{H_{38}}$-***b***-$\mathbf{G4_{75}}$ (M_n=30.6 kDa) at 1.5 mg mL^{-1} (corresponding to [HIABMA]=1.9x10^{-3}M) with HCl=0.1 N and starting with ~50% excess of NaOH (addition rate= 0.01 mL min^{-1}).

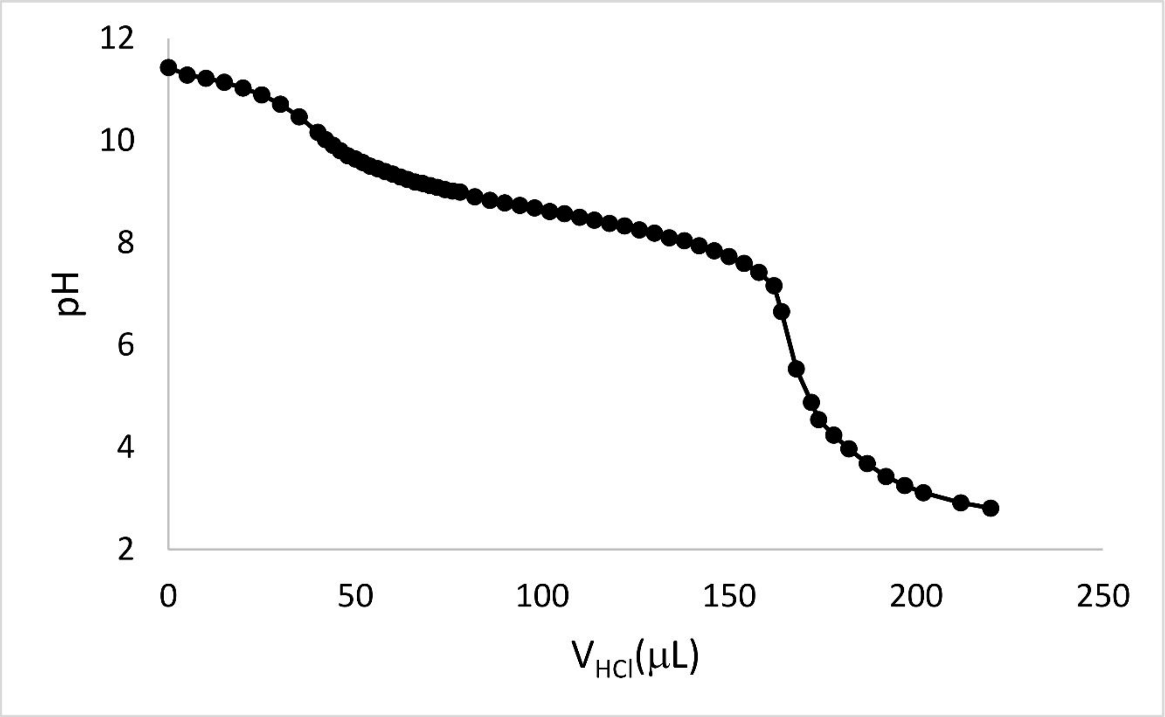

Figure S6. Titration curve of HIABMA monomer (3.0 mg, 0.014 mmol) at 4.9×10^{-3} M with HCl=0.1 N and starting with ~20% excess of NaOH (addition rate= 0.01 mL min^{-1}).

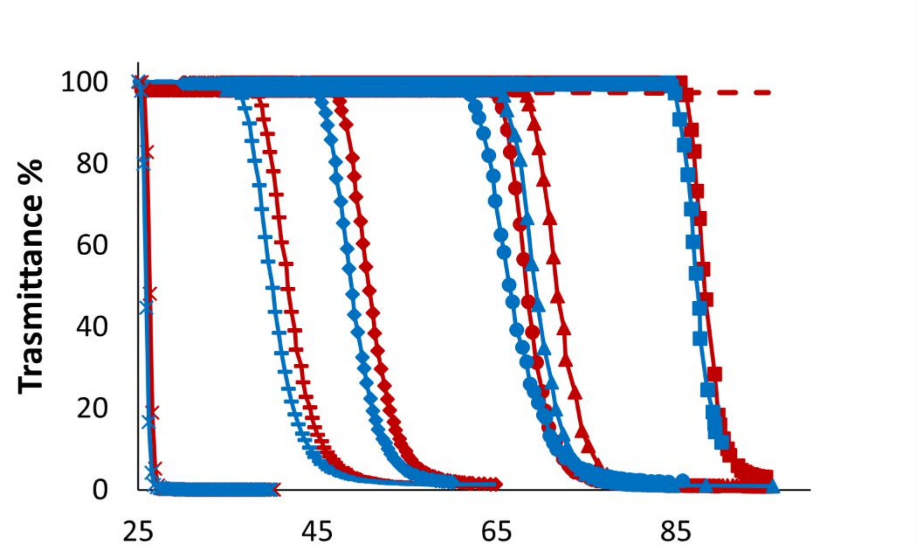

Figure S7. Plots of transmittance as a function of temperature (700 nm, 1 °C min^{-1}) measured for aqueous solutions of $\mathbf{GX_m}$ and $\mathbf{G2_mG9_n}$ polymers. Red lines: Heating cycles; blue lines: Cooling cycles. (x) $\mathbf{G2_{75}}$ (3.0 mg mL^{-1}); (-) $\mathbf{G2_{69}G9_6}$ (0.75 mg mL^{-1}); (◆) $\mathbf{G2_{64}G9_{11}}$ (0.8 mg mL^{-1}); (●) $\mathbf{G2_{53}G9_{22}}$ (1.1 mg mL^{-1}); (▲) $\mathbf{G4_{75}}$ (1.1 mg mL^{-1}); (□) $\mathbf{G2_{22}G9_{53}}$ (1.02 mg mL^{-1}); (dashed red line) $\mathbf{G9_{75}}$ (1 mg mL^{-1}).

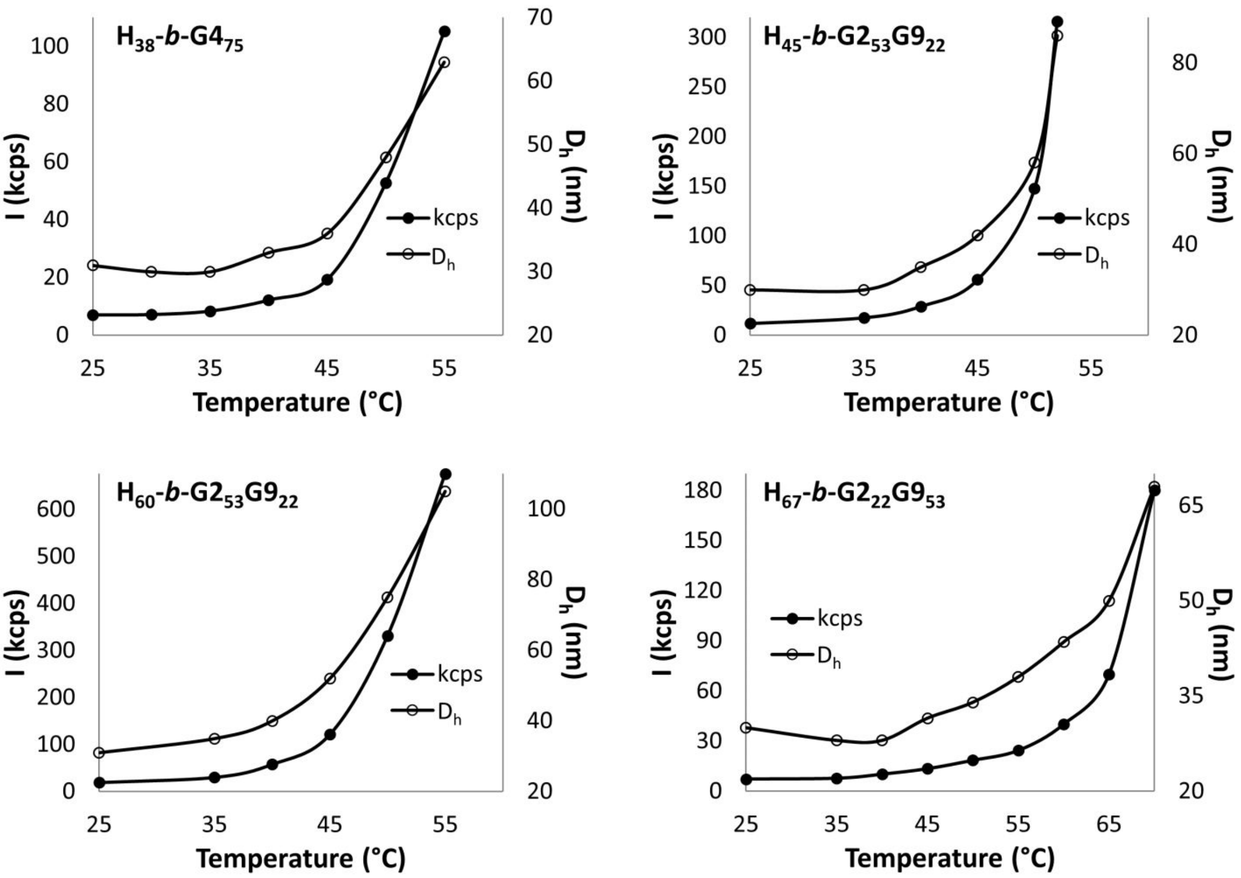

Figure S8. kcps and D_h (DLS) as a function of temperature of 1.5 mg mL^{-1} PIM micelles in water.

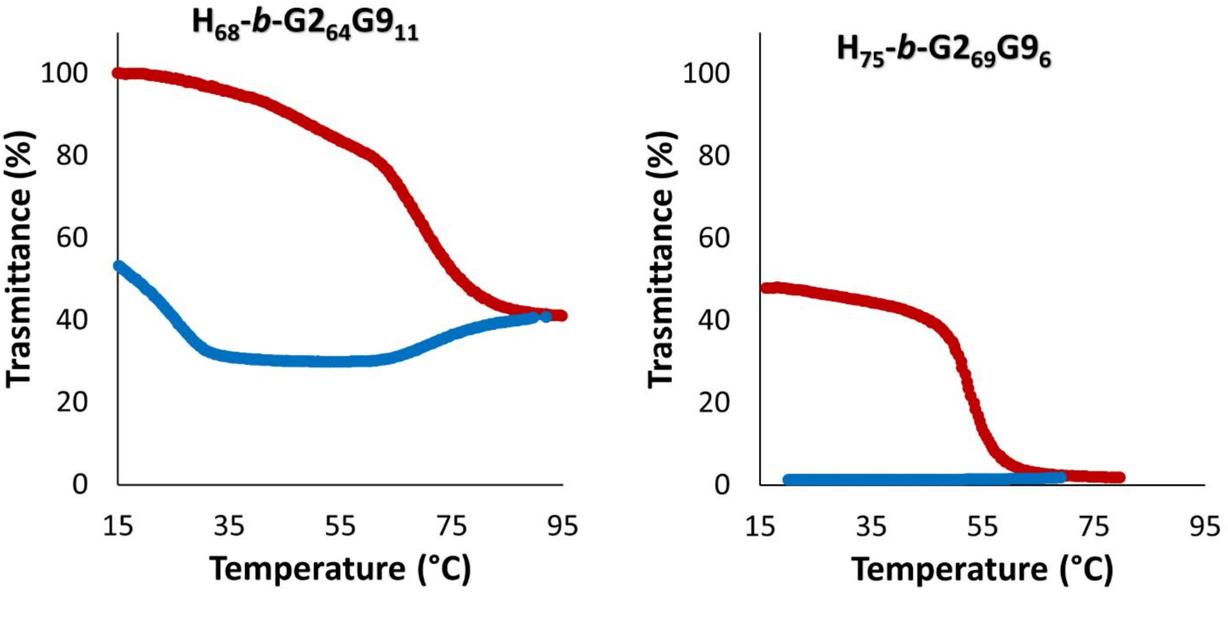

Figure S9. Plots of transmittance as a function of temperature (700 nm, 1 °C min^{-1}) of 1.5 mg mL^{-1} PIM micelles in water. Red lines: heating cycles; Blue lines: cooling cycles.

Table S1 Molar masses (MW), molecular volumes (V), partial specific volumes ($\tilde{\upsilon}$), electron content (Z) and scattering length densities (SLD) estimated for the polymers analyzed by SAXS and their PHIABMA (H) and POEGMA (**GX**, with X=4,9 or 2) blocks.

	MW[a] (g/mol)	V[b] (nm^3)	$\tilde{\upsilon}$ (cm^3/g)	Z[a] (electrons)	SLD (10^{10} cm^{-2})
**H_{38}-*b*-$G4_{75}$**	29048	40.5	0.840	15698	10.92
H_{38} block	8256	11.0	0.802	4411	11.30
$G4_{75}$ block	20792	29.5	0.855	11287	10.77
**H_{93}-*b*-$G9_{75}$**	57295	79.7	0.837	30968	10.96
H_{93} block	19983	26.6	0.803	10681	11.30
$G9_{75}$ block	37312	53.0	0.856	20287	10.78
**H_{60}-*b*-$G2_{53}G9_{22}$**	33915	47.0	0.835	18302	10.96
H_{60} block	12947	17.3	0.803	6919	11.30
$G2_{53}G9_{22}$ block	20968	29.8	0.855	11383	10.77
H_2O	18.01	0.0300[c]	1.002[c]	10	9.40
DMF	73.09	0.1285[d]	1.059[d]	40	8.77

[a] The molecular mass and the number of electrons were calculated from the reported structural formulas (**Scheme 1** in the main text).

[b] The volumes were estimated using the empirical method of Durchschlag, Zipper.[2]

[c] From water density at 25°C.[3]

[d] From dimethylformamide density at 25°C.[4]

Table S2. Dimensional parameters obtained by SAXS analysis of the copolymer **H_{93}-*b*-$G9_{75}$** across the solvent composition (water weight % in water-DMF mixtures) trend realized in the Solvent Displacement protocol for micelle formation (data shown in Fig. SAXS1). SAXS analysis was also performed on samples diluted to the same final concentration of 2 mg mL^{-1} to highlight the solvent effect over possible concentration effects. Due to the different solvent composition, the contrast of scattering length per mass of polymer (ΔSL_m) used in the estimate of the aggregate molecular weight (MW) and aggregation number (N_{agg}) from the I(0) values is also reported.

wt% H_2O	c ($mgmL^{-1}$)	R_g (nm)	D_{max} (nm)	I(0) (cm^{-1})	ΔSL_m[b] (10^{10}cm/g)	MW (kDa)	N_{agg}
0	10	5.1 ±0.1	18 ±1	0.242 ±0.003	1.83	44 ±0.6	1 ±0.01
32	6.9	5.7 ±0.1	17 ±1	0.183 ±0.002	1.44	77 ±0.8	1 ±0.01
40	6.1	15.5 ±0.1	50 ±2	7.47 ±0.05	1.39	3809 ±24	66 ±0.4
80	2.1	12.0 ±0.1	37 ±2	2.27 ±0.02	1.33	3696 ±27	65 ±0.2
100[a]	1.5	11.4 ±0.1	36 ±2	1.48 ±0.01	1.30	3510 ±21	61 ±0.4
0	2	4.4 ±0.1	13 ±2	0.043 ±0.001	1.83	38 ±1	1 ±0.02
32	2	5.9 ±0.3	20 ±2	0.071 ±0.004	1.44	111 ±6	2 ±0.1
40	2	15.8 ±0.2	50 ±2	2.06 ±0.02	1.39	3522 ±37	61 ±0.7

[a] after dialysis of the 80 wt% sample in water.

[b] from the SLD values estimated for the polymers (**Table S1**) and those of DMF-water mixtures according to reported density values.[4]

Table S3. Details of the models and parameters used for obtaining the calculated SAXS profiles shown in Figure 2 (a,f), Figure S10 (b), Figure 6 (c,d), Figure 7 (d,e,g,h).

a) Sample	H_{93}-*b*-$G9_{75}$ 10 mg mL^{-1} in DMF	
Model	Generalized gaussian coil (SASfit 0.94.11 documentation, model 8.4.1.5)	
q range (nm^{-1})	0.115-5	
reduced χ^2	2.086	
Parameters		
background (cm^{-1})	0.000089	Optimized
R_g (radius of gyration, nm)	5.25 ± 0.11	Optimized
ν (excluded volume parameter)	0.45 ± 0.02	Optimized
I(0) (cm^{-1})	0.243 ± 0.008	Optimized
N (number density, cm^{-3})	1.05□10^{17}	$N = \frac{c[g/cm^3]N_{Avogadro}[1/mol]}{MW[g/mol]}$
V (polymer volume, nm^3)	82.2	$I(0) = N\frac{d\sigma}{d\Omega} = NV^2(r_{Th}^2\frac{Z_{polymer}}{V} - SLD_{solvent})^2$

b) Sample	H_{93}-*b*-$G9_{75}$ 2 mg mL^{-1} in DMF	
Model	Generalized gaussian coil (SASfit 0.94.11 documentation, model 8.4.1.5)	
q range (nm^{-1})	0.045-5	
reduced χ^2	1.435	
Parameters		
background (cm^{-1})	0.000016	Optimized
R_g (radius of gyration, nm)	5.52 ± 0.09	Optimized
ν (excluded volume parameter)	0.48 ± 0.23	Optimized
I(0) (cm^{-1})	0.0505 ± 0.04	Optimized
N (number density, cm^{-3})	2.10□10^{16}	$N = \frac{c[g/cm^3]N_{Avogadro}[1/mol]}{MW[g/mol]}$
V (polymer volume, nm^3)	81.8	$I(0) = N\frac{d\sigma}{d\Omega} = NV^2(r_{Th}^2\frac{Z_{polymer}}{V} - SLD_{solvent})^2$

c) Sample	H_{38}-*b*-$G4_{75}$ 1.5 mg mL^{-1} pH 11	
Model	Generalized gaussian coil (SASfit 0.94.11 documentation, model 8.4.1.5)	
q range (nm^{-1})	0.045-6.3	
reduced χ^2	1.260	
Parameters		
Background (cm^{-1})	0.000079	Optimized
R_g (radius of gyration, nm)	2.7 ± 0.1	Optimized
ν (excluded volume parameter)	0.35 ± 0.17	Optimized
I(0) (cm^{-1})	0.019 ± 0.002	Optimized
N (number density, cm^{-3})	3.11×10^{16}	$N = \frac{c[g/cm^3] N_{Avogadro}[1/mol]}{MW[g/mol]}$
V (polymer volume, nm^3)	38.8	$I(0) = N\frac{d\sigma}{d\Omega} = NV^2(r_{Th}^2\frac{Z_{polymer}}{V} - SLD_{solvent})^2$

d) Sample	H_{38}-*b*-$G4_{75}$ 1.5 mg mL^{-1} pH 7.3 (PIM)	
Model	Block copolymer micelle (SASfit 0.94.11 documentation, model 3.2.6)	
q range (nm^{-1})	0.045-7	
reduced χ^2	1.393	
Parameters		
N_{agg} (aggregation number)	48.1	Fixed
$V_{block\ core}$ (nm^3)	11	Fixed
$V_{block\ shell}$ (nm^3)	29.5	Fixed
SLD_{core} (nm^{-2})	11.3×10^{-4}	Fixed
SLD_{shell} (nm^{-2})	10.78×10^{-4}	Fixed
$SLD_{solvent}$ (nm^{-2})	9.40×10^{-4}	Fixed
d (penetration parameter)	1	Fixed
R_g (nm)	3.24 ± 0.03	Optimized
N (number density, nm^{-3})	6.20×10^{-7}	Optimized

Background (cm^{-1})	0.00025	Optimized
R_{core} (nm)	5.0	Calculated $R_{core} = \sqrt[3]{\frac{3N_{agg}V_{core}}{4\pi}}$
σ (surface coverage index)[5]	1.853	Calculated $\sigma = \frac{\pi R_g^2 N_{agg}}{4\pi(R_{core} + R_g)^2}$

e) Sample	H_{93}-*b*-$G9_{75}$ 1.5 mg mL^{-1} pH 7.2 (PIM)	
Model	Block copolymer micelle (SASfit 0.94.11 documentation, model 3.2.6)	
q range (nm^{-1})	0.085-7	
reduced χ^2	2.811	
Parameters		
N_{agg} (aggregation number)	24.3	Fixed
$V_{block\ core}$ (nm^3)	26.6	Fixed
$V_{block\ shell}$ (nm^3)	53.0	Fixed
SLD_{core} (nm^{-2})	11.3□10^{-4}	Fixed
SLD_{shell} (nm^{-2})	10.78□10^{-4}	Fixed
$SLD_{solvent}$ (nm^{-2})	9.40□10^{-4}	Fixed
d (penetration parameter)	1	Fixed
R_g (nm)	2.95 ± 0.03	Optimized
N (number density, nm^{-3})	5.87□10^{-7}	Optimized
Background	0.000014	Optimized
R_{core} (nm)	5.4	Calculated $R_{core} = \sqrt[3]{\frac{3N_{agg}V_{core}}{4\pi}}$
σ (surface coverage index)	0.764	Calculated $\sigma = \frac{\pi R_g^2 N_{agg}}{4\pi(R_{core} + R_g)^2}$

f) Sample	H_{93}-*b*-$G9_{75}$ 1.5 mg mL^{-1} SD
Model	Block copolymer micelle (SASfit 0.94.11 documentation, model 3.2.6)
q range (nm^{-1})	0.045-6

reduced χ^2	1.394	
Parameters		
N_{agg} (aggregation number)	61.3	Fixed
$V_{block\ core}$ (nm^3)	26.6	Fixed
$V_{block\ shell}$ (nm^3)	53.0	Fixed
SLD_{core} (nm^{-2})	11.3□10^{-4}	Fixed
$SLD_{solvent}$ (nm^{-2})	9.40□10^{-4}	Fixed
SLD_{shell} (nm^{-2})	10.41 ± 0.02 □10^{-4}	Optimized
x_{core} (volume fraction solvent)	0.43 ± 0.02	Optimized
d (penetration parameter)	1	Fixed
R_g (nm)	4.15 ± 0.01	Optimized
N (number density, nm^{-3})	3.69□10^{-7}	Optimized
Background	0.000061	Optimized
R_{core} (nm)	8.8	Calculated $R_{core} = \sqrt[3]{\frac{3N_{agg}V_{core}}{(1-x_{core})4\pi}}$
σ (surface coverage index)	1.567	Calculated $\sigma = \frac{\pi R_g^2 N_{agg}}{4\pi(R_{core}+R_g)^2}$

g) Sample	H_{60}-*b*-$G2_{53}G9_{22}$ 10 mg mL^{-1} pH 6 (PIM)	
Model	Block copolymer micelle (SASfit 0.94.11 documentation, model 3.2.6)	
q range (nm^{-1})	0.045-7	
reduced χ^2	505 (disagreement)	
Parameters		
N_{agg} (aggregation number)	97.4	Fixed
$V_{block\ core}$ (nm^3)	17.3	Fixed
$V_{block\ shell}$ (nm^3)	29.8	Fixed
SLD_{core} (nm^{-2})	11.3□10^{-4}	Fixed

SLD_{shell} (nm^{-2})	10.78□10^{-4}	Fixed
$SLD_{solvent}$ (nm^{-2})	9.40□10^{-4}	Fixed
d (penetration parameter)	1	Fixed
R_g (nm)	3.53 ± 0.01	Optimized
N (number density, nm^{-3})	1.80□10^{-6}	Optimized
Background	0.00085	Optimized

h) Sample	H_{60}-*b*-$G2_{53}G9_{22}$ 10 mg mL^{-1} pH 6 (PIM)	
Model	Sphere+Chains(RandomWalk)_Rc (SASfit 0.94.11 documentation, model 3.2.4.2)	
q range (nm^{-1})	0.045-7	
reduced χ^2	1.877	
Parameters		
$SLD_{solvent}$ (nm^{-2})	9.40□10^{-4}	Fixed
SLD_{shell} (nm^{-2})	10.78□10^{-4}	Fixed
SLD_{core} (nm^{-2})	11.05□10^{-4}	Fixed (estimated as H_{60} block+ $G2_{53}$ sub-block)
$\langle R_{core} \rangle$(nm)	10.3	Optimized
σ_{Rcore}(Lognorm distribution)	0.15	Optimized
$V_{block\ core}$ (nm^3)	47.3	Optimized
$V_{block\ shell}$ (nm^3)	20.6	Optimized
x_{core} (volume fraction solvent)	0.486	Optimized
d (penetration parameter)	0.35	Optimized
R_g (nm)	2.37	Optimized
N (number density, nm^{-3})	2.21□10^{-6}	Optimized
Background	0.00072	Optimized

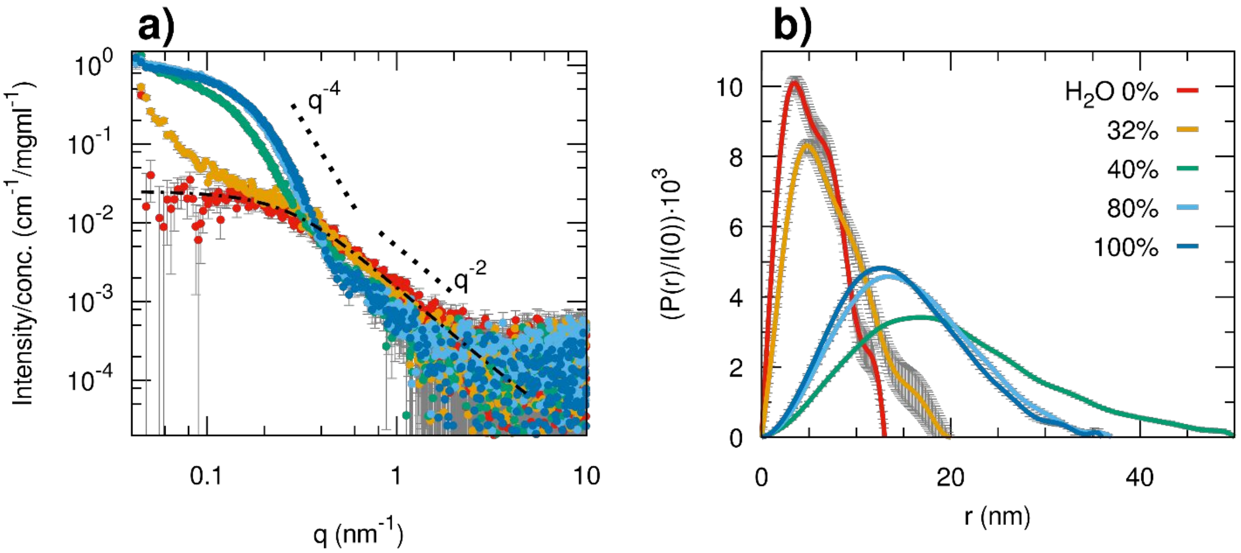

Figure S10. a) SAXS data of $\mathbf{H_{93}}$-***b*****-**$\mathbf{G9_{75}}$ at different solvent compositions (water weight % in water-DMF mixtures) explored in the Solvent Displacement method for obtaining micellar aggregates. Compared to Figure SAXS1, here the SAXS data for samples with water content < 80% were measured on samples diluted in the same water-DMF mixture to 2 mg mL^{-1} to be at a similar concentration as the final micellar sample. The data at 0% are fitted with the analytical model of a generalized Gaussian coil (dot-dashed black line)[6] whose parameters are reported in **Table S3b**; b) Pair distance distribution functions P(r) obtained by indirect Fourier transform of the SAXS data in a).

Bibliography

(1) B. J. Berne and R. Pecora, *Dynamic Light Scattering: With Applications to Chemistry*, Biology and Physics, Dover Books, New York, **2000**; W. Schärtl, *Light Scattering from Polymer Solutions and Nanoparticle Dispersions*, Springer, Berlin, **2007.**

(2) Durchschlag, H.; Zipper, P. *Calculation of the Partial Volume of Organic Compounds and Polymers*; Springer-Verlag GmbH & Company KG, 1994; Vol. 94. https://doi.org/10.1007/bfb0115599.

(3) Lemmon, E. W.; McLinden, M. O.; Friend, D. G. Thermophysical Properties of Fluid Systems. In *NIST Chemistry WebBook, NIST Standard Reference Database NUmber 69*; Linstrom, P. J., Mallard, W. G., Eds.; National Institute of Standards and Technology: Gaithersburg MD, 2020. https://doi.org/10.18434/T4D303.

(4) Ueno, M.; Mitsui, R.; Iwahashi, H.; Tsuchihashi, N.; Ibuki, K. Pressure and Temperature Effects on the Density and Viscosity of DMF-Water Mixtures. In *Journal of Physics: Conference Series*; 2010; Vol. 215, p 012074. https://doi.org/10.1088/1742-6596/215/1/012074.

(5) Svaneborg, C.; Pedersen, J. S. Form Factors of Block Copolymer Micelles with Excluded-Volume Interactions of the Corona Chains Determined by Monte Carlo Simulations. *Macromolecules* **2002**, *35* (3), 1028–1037. https://doi.org/10.1021/ma011046v.

(6) Hammouda, B. SANS from Homogeneous Polymer Mixtures: A Unified Overview. *Advances in Polymer Science*. Springer New York 1993, pp 86–133. https://doi.org/10.1007/bfb0025862.
